# Macrophages expressing macrophage receptor with collagen structure attenuate liver fibrosis through a tissue restoration phenotype

**DOI:** 10.1172/jci.insight.193172

**Published:** 2026-03-23

**Authors:** Sofia Jerez, Shawna A. Cooper, Usman Yaqoob, Maleeha F. Kalaiger, Abid A. Anwar, Mandy Wong, Bushra Arif, Luke C. Doskey, Maria Hernandez-Tejero, William A. Sherman, Ruben De Boeck, Ying Li, Moira B. Hilscher, Enis Kostallari, Nidhi Jalan-Sakrikar, Sheng Cao, Vijay H. Shah

**Affiliations:** 1 Division of Gastroenterology and Hepatology,; 2 Graduate School of Biomedical Sciences, Mayo Clinic College of Medicine and Science,; 3 Department of Biochemistry and Molecular Biology,; 4 Graduate Research Education Program, and; 5 Department of Quantitative Health Sciences, Mayo Clinic, Rochester, Minnesota, USA.; 6 Department of Quantitative Health Sciences, Mayo Clinic, Jacksonville, Florida, USA.

**Keywords:** Cell biology, Hepatology, Fibrosis, Immunotherapy, Macrophages

## Abstract

Liver macrophages are central in maintaining hepatic homeostasis and mediating immune responses during liver injury, including fibrosis. Macrophages may have proinflammatory or antiinflammatory properties, but which properties influence fibrosis remains unclear. To explore the role of macrophages in liver fibrosis, we performed single-cell RNA-seq in a mouse model of liver injury and found that macrophage diversity was increased. *Marco* was among the most significantly upregulated genes, and a population of *Marco*^hi^ macrophages increased with injury and spatially segregated to nonfibrotic areas. The macrophage receptor with collagenous structure (MARCO) protein is a scavenger receptor expressed by specific subsets of macrophages, and its role in liver fibrosis is unclear. In vitro induction of *Marco* in bone marrow–derived macrophages decreased proinflammatory gene expression, increased antiinflammatory and antifibrotic gene expression, and enhanced phagocytosis, indicating a restorative phenotype. Adoptive transfer of MARCO^+^ macrophages in a mouse model of liver fibrosis reduced the expression of extracellular matrix–associated (ECM-associated) genes in hepatic stellate cells (HSCs) and reduced collagen deposition, which did not occur with the transfer of MARCO^–^ macrophages. Therefore, MARCO^+^ macrophages have a tissue restorative role in the liver and attenuate fibrogenesis through interaction with HSCs, thereby providing a potential therapeutic pathway for liver fibrosis.

## Abstract 2

Injection of bone marrow-derived macrophages (BMDM) expressing Macrophage receptor with a collagenous structure (MARCO) reduces scarring in a mouse model of chronic liver fibrosis.

## Graphical abstract

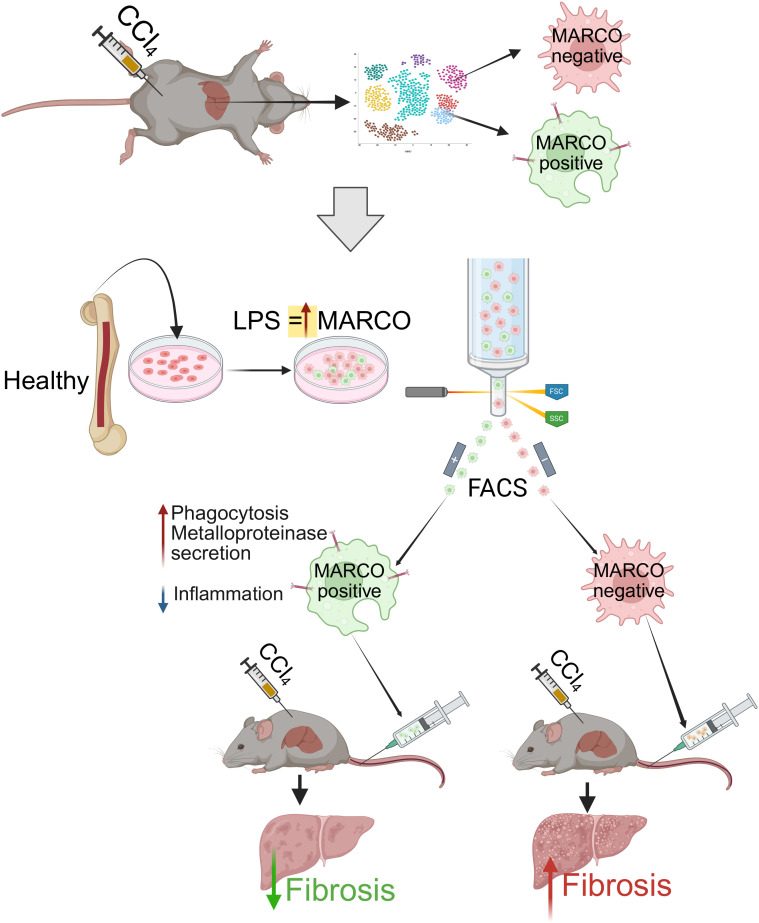

## Introduction

Chronic liver diseases are characterized by excessive intrahepatic inflammation, which leads to the development of fibrosis, cirrhosis, and liver failure with an increased risk of death (1). Fibrosis is characterized by the accumulation of extracellular matrix (ECM) and subsequent loss of liver function (1, 2). Additionally, cytokine and chemokine secretion are increased, which promotes the infiltration of immune cells, including macrophages, to the liver (1). The liver hosts the largest population of macrophages in the body (3, 4); however, the role of macrophages in liver disease is not fully understood. In healthy livers, there is a homeostatic balance between resident Kupffer cells and infiltrating monocyte-derived macrophages (5). The normal functions of these macrophage subpopulations include regulating homeostasis, monitoring for pathogens, and clearing debris generated during normal apoptotic processes of hepatic cells. However, the heterogeneity and distribution of macrophage subpopulations during liver injury and fibrosis remain unclear.

The macrophage receptor with collagenous structure (MARCO) is a class A membrane protein member of the scavenger receptor family, which is primarily expressed by macrophages (6, 7). With recent advances in single-cell technologies, MARCO^+^ macrophages have been increasingly detected in multiple tissues, and their potential role in health and disease has become an area of interest for multiple organ systems (8). MARCO protein expression is associated with an immunosuppressive phenotype during tumorigenesis (9–11) and has been proposed as an immune checkpoint mediator that contributes to tumor cell immune evasion (12). MARCO protein is also upregulated in fibrotic skin and lung diseases (13–15). Additionally, proinflammatory cytokine production is potentially decreased in MARCO^+^ macrophages (16, 17). However, the role of MARCO^+^ macrophages in liver fibrosis remains unknown. The function of MARCO^+^ macrophages in tissues other than the liver includes recognition and clearance of pathogens, apoptotic cells, and debris, which suggests that MARCO^+^ macrophages may contribute to liver health and function (18, 19).

Cell-based therapy is an emerging approach for treating diseases for which specific pharmacologic therapies are not available or effective. Ex vivo–polarized immune cells or engineered immune cells, such as chimeric antigen receptor T cells, are increasingly used to treat chronic conditions in addition to cancer (20, 21). The phenotypic plasticity of macrophages makes them attractive candidates for cell-based therapy. Indeed, macrophage-based treatment for tissue repair and regeneration has been reported in animal models of several nonliver conditions, including autoimmune encephalomyelitis (22), spinal cord injury (23), and tendon repair (24). Similarly, macrophage-based therapy has been used to restore immune homeostasis and reduce inflammation in autoimmune diseases such as multiple sclerosis and rheumatoid arthritis (25). However, few macrophage-based therapies have been developed to treat liver fibrosis (26). Therefore, understanding the role of MARCO^+^ macrophages in liver diseases may yield insight into developing targeted therapies to modulate immune responses in the liver.

## Results

### Chronic liver fibrosis induces transcriptomic changes in macrophage subpopulations.

**Figure 1:**
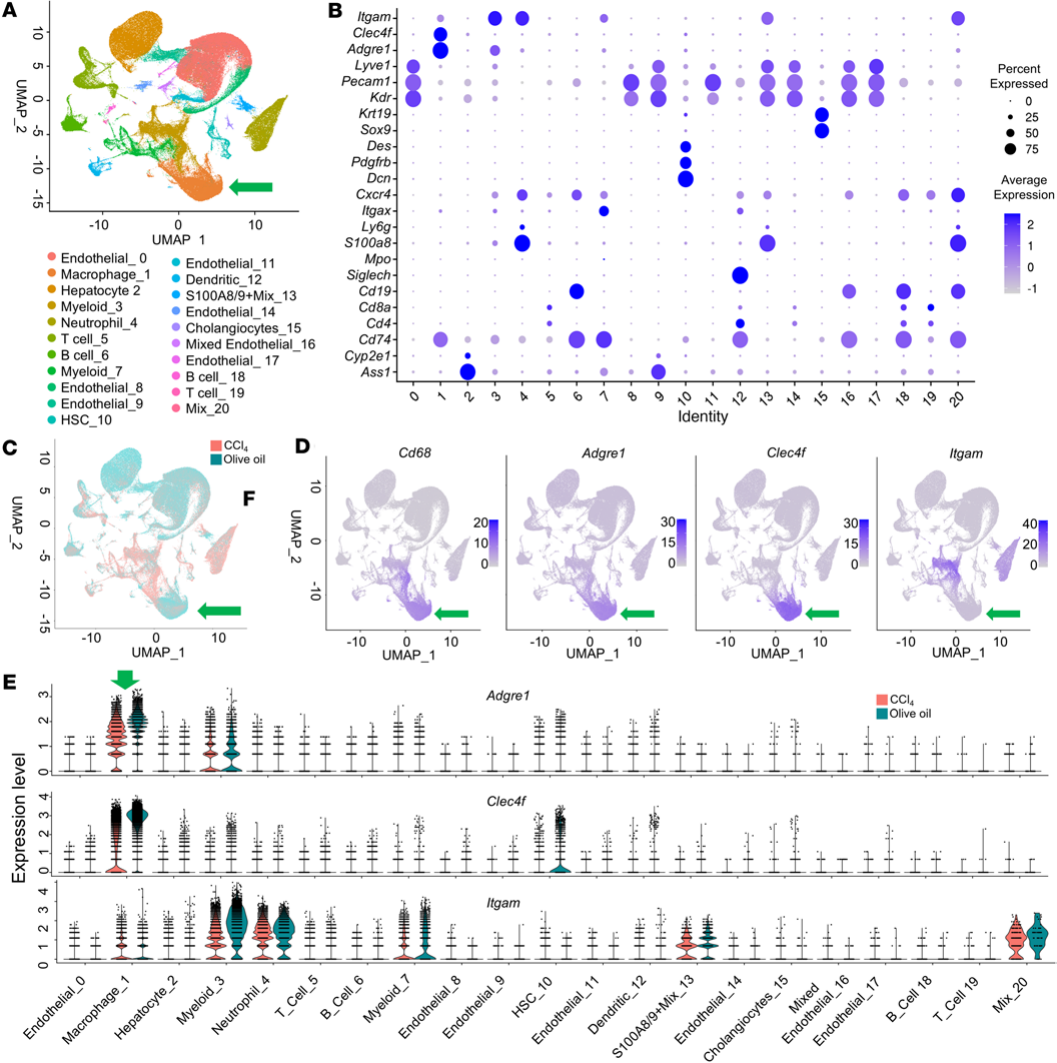
scRNA-seq identifies 2 predominant macrophage populations after CCl_4_-induced fibrosis. (**A**) Unsupervised clustering yielded 21 major clusters at 0.1 resolution, which were identified to cell type according to expression of the established cell marker genes shown in the dot plot in **B**. Each dot represents a single cell. One clean macrophage cluster was used for downstream analysis (with green arrow). (**B**) Dot plot of established cell marker genes to identify the cell type of each cluster shown in **A**. (**C**) UMAP plot highlights the sample distribution between liver cells isolated from mice after olive oil or CCl_4_ administration. (**D**) Feature plots depicting expression of macrophage marker genes in clusters 1 and 3 (green arrow): general markers *Cd6*8 and *Adgre1* (F4/80 coding gene); Kupffer cell marker *Clec4*f, and infiltrating macrophage marker *Itgam* (CD11B coding gene). (**E**) Violin plots of differential gene expression showing expression of macrophage identity genes in the correct clusters. *Adgre*1 indicates adhesion G protein-coupled receptor E1.

To assess the effect of chronic fibrosis on the macrophage population and transcriptomic diversity, single-cell RNA-seq (scRNA-seq) was performed on intrahepatic leukocytes isolated from 8 mice given olive oil (control) or CCl_4_ (chronic fibrosis). Bioinformatic quality control focused on recovering high-quality, singlet nonparenchymal cells rather than hepatocytes. This yielded 139,159 cells from the olive oil group and 107,366 from the CCl_4_ group across 21 clusters at 0.1 resolution (Figure 1, A–C). Cluster identity was determined by canonical cell-type conserved genes (Figure 1B).

Multiple myeloid clusters were identified, but macrophage marker genes, such as the F4/80-coding gene *Adgre1*, *Clec4f*, and *Itgam*, identified 2 potential macrophage clusters: cluster 1 (Olive Oil: 16,641, CCl_4_: 11,017), and cluster 3 (Olive Oil: 2,874, CCl_4_: 12,557) (Figure 1B). Macrophage subpopulations were annotated by using conserved genes, with cluster 1 determined to be primarily composed of Kupffer cells expressing *Adgre1* and *Clec4f* (Figure 1, D and E) with a minority of infiltrating monocyte-derived macrophages, which were identified by the expression of the myeloid cell marker CD11B-coding gene *Itgam* (Figure 1, D and E) also present. Cluster 3 was positive for *Adgre1* and *Itgam*, but also for genes suggesting a mixture of nonmacrophage myeloid cell types and endothelial cells.

The data show that, at this resolution, we can identify the 2 predominant macrophage populations in olive oil and CCl_4_ groups, resident Kupffer cells and infiltrating monocyte-derived macrophages.

### Marco expression in macrophages is enhanced during chronic liver fibrosis.

**Figure 2:**
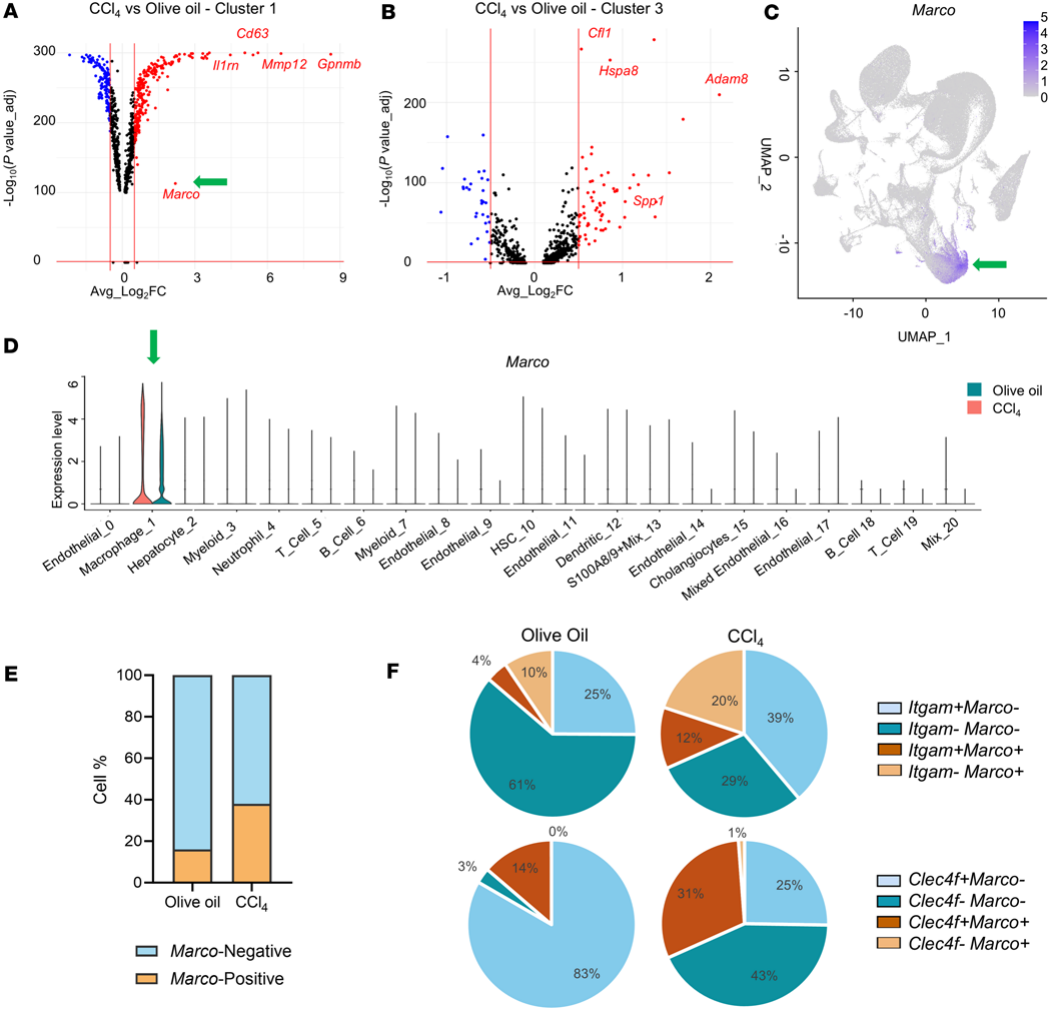
Expression of *Marc*o is upregulated in a specific macrophage subpopulation in CCl_4_-induced chronic fibrosis. (**A**) Volcano plot of differential gene expression between macrophages in cluster 1 for liver cells isolated from mice after olive oil or CCl_4_ administration. Downregulated genes are shown in blue, upregulated genes in red, and unchanged genes in black. Arrow shows *Marc*o as one of the genes with the greatest upregulated expression in this cluster. (**B**) Volcano plot of differential gene expression between macrophages in cluster 3 for liver cells isolated from mice after olive oil or CCl_4_ administration. Downregulated genes are shown in blue, upregulated genes in red, and unchanged genes in black. This cluster failed to show a clear enrichment for proinflammatory macrophages and appeared to be somewhat mixed, so it was not utilized for downstream analysis. (**C**) Feature plot of *Marco* expression restricted to the macrophage cluster as shown in Figure 1, A and E. (**D**) Violin plot of *Marc*o differential expression in cluster 1 liver cells isolated from mice after olive oil or CCl_4_ administration. Note that *Marco* expression is restricted to cluster 1. (**E**) Bioinformatic subsetting and sorting of *Marc*o^+^ cells demonstrates that the proportion of *Marc*o^+^ cells nearly doubled with CCl_4_-induced fibrosis. (**F**) Bioinformatic subsetting and sorting for coexpression of *Marc*o and the Kupffer cell marker *Clec4f* versus infiltrating macrophage marker *Itgam* demonstrates that *Marc*o^+^ cells are almost exclusively *Clec4f*^+^ (Kupffer cells) in the healthy olive oil condition but expand to include an *Itgam^+^Marco*^+^ population with CCl_4_-induced fibrosis. Differential gene expression was calculated on scTransform v2 normalized data with FindMarkers using the Wilcoxon rank sum test and reported as average log_2_ fold change and Benjamini-Hochberg–adjusted *P* values.

To understand the transcriptional profiles of each of the macrophage subpopulations during CCl_4_-induced liver fibrosis, differential gene expression analysis was conducted. For each of the identified transcriptomic clusters, differentially expressed genes in the livers of the olive oil group versus CCl_4_ group were identified by using a cutoff value of log_2_FC change greater than 0.5 and an adjusted *P* value less than 0.05. In cluster 1, the most highly upregulated genes included *Gpnmb*, *Cd63*, and *Marco*. *Marco* was one of the highly upregulated genes in the CCl_4_ group, with a log_2_ fold change of 2.2 and an adjusted *P* value of 1 × 10^–113^ (Figure 2A). *Marco* is known to be expressed on tissue-resident macrophages in the lung and liver (5, 27); concordantly, in our study, *Marco* was expressed in the Kupffer cell cluster 1, as shown by violin and feature plots of gene expression (Figure 2, C and D, and Supplemental Figure 1; supplemental material available online with this article; https://doi.org/10.1172/jci.insight.193172DS1). Despite low levels of expression in the olive oil group, in the livers of CCl_4_-induced fibrotic mice, the proportion of macrophages expressing *Marco* doubled to over 30% (Figure 2E and Supplemental Figure 2), indicating that its expression is upregulated in response to chronic fibrosis.

To better understand the phenotypes and origins of liver macrophages expressing *Marco*, we focused on cluster 1 to avoid the potential for bias from cluster 3. Cluster 3 was highly unbalanced for cell counts between olive oil and CCl_4_ and presented with a heterogeneous population of NPC fraction. Within cluster 1, refined subpopulations of cells were obtained for determining their cellular origin, differential gene expression, and downstream analysis. During CCl_4_-induced fibrosis, there was an increase of the infiltrating *Itgam^+^* and *Clec4f^–^* macrophages in the liver, and in addition to the resident *Clec4f^+^Marco^+^* cells; *Itgam^+^Marco^+^* *and Clec4f^–^Marco^+^* macrophage populations emerge (Figure 2F), suggesting that *Marco* expression is also increased on smaller populations of infiltrating macrophages during liver fibrosis. To further refine this population, the cells were triple-sorted for *Clec4f*, *Itgam*, and *Marco* to attempt to identify infiltrating myeloid cells that adopt the Kupffer cell phenotype (moKC) versus embryonic-derived Kupffer cells (Supplemental Figure 2). Indeed, the proportion of cells positive for *Clec4f^+^* and negative for *Itgam^–^* and *Marco^–^* decreased from 60% in the Olive Oil group to 13% in the CCl_4_. This was coupled with an increase in *Clec4f^+^Itgam^–^Marco^+^* (likely embryonic-derived KCs) from 10% in Olive Oil to 19% in CCl_4_ as well as an increase in the triple-positive group *Clec4f^+^Itgam^+^Marco^+^* (likely moKC) from 4% to 11%. This suggests that increases in injury-induced *Marco* expression are occurring in heterogeneous populations of Kupffer cells.

Next, differential gene expression analysis and IPA (Qiagen) were conducted on *Marco*^+^ versus *Marco*^–^ macrophages from the olive oil group and CCl_4_ group (Supplemental Figure 3, A and B, and Supplemental Figure 4) and between *Marco*^+^ macrophages from olive oil versus CCl_4_ (Supplemental Figure 3C). These analyses showed that phagosome formation (phagocytosis) pathway genes increased in *Marco*^+^ macrophages compared with *Marco*^–^ macrophages from the olive oil group control and CCl_4_-induced fibrosis livers. However, phagocytosis pathway genes were decreased in *Marco*^+^ macrophages in CCl_4_-induced fibrosis livers compared with *Marco*^+^ macrophages from olive oil. These results suggest that the *Marco*^+^ macrophages could be more functional in their ability to participate in ECM remodeling and fibrosis prevention than *Marco*^–^ macrophages, but that after CCl_4_-induced fibrosis, this capability is attenuated in *Marco*^+^ macrophages.

### Pseudotime analysis indicates that Marco^+^ macrophages originate from Kupffer cells and infiltrating macrophages in CCl_4_ fibrosis.

**Figure 3:**
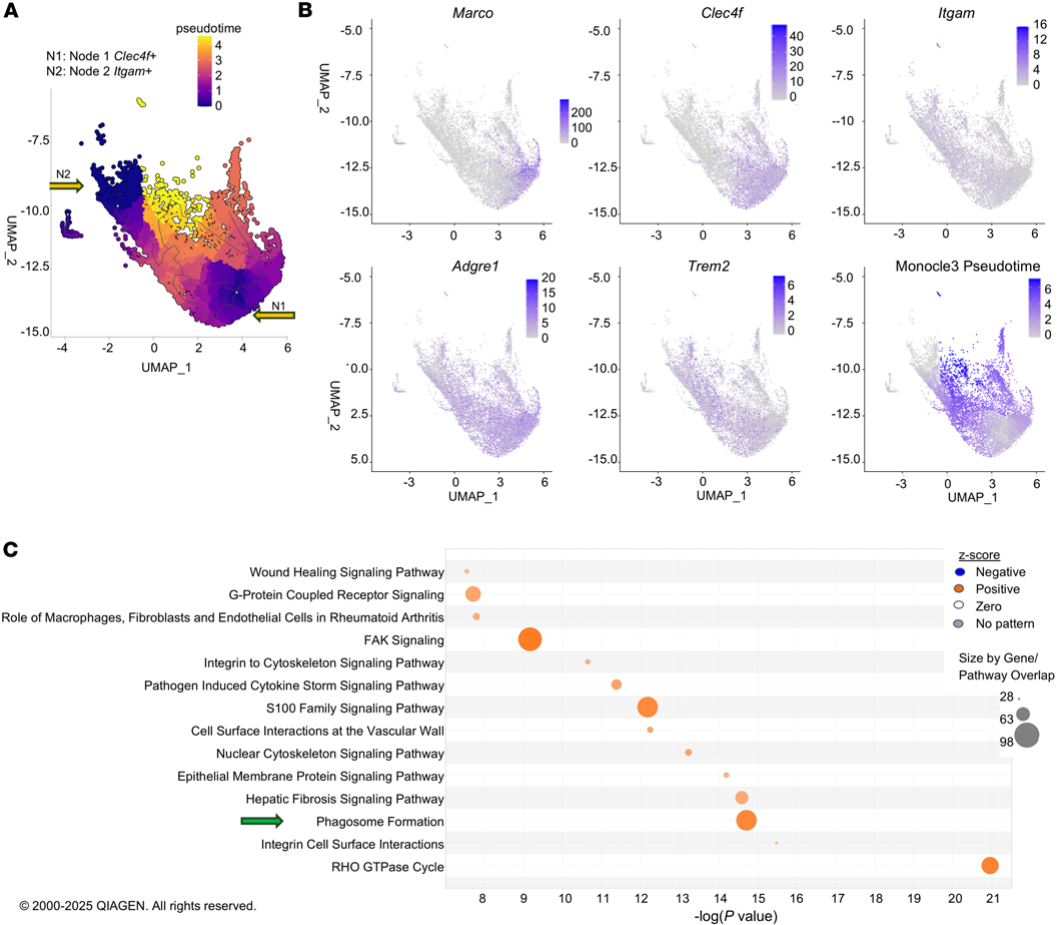
Pseudotime analysis of macrophage phenotypic plasticity in chronic CCl_4_-induced fibrosis. (**A**) Trajectory analysis of the CCl_4_ macrophage population with Monocle 3 software (63), with a starting node rooted in the *Clec4f*^+^ macrophage population and *Itgam^+^* population (primarily Kupffer cells and infiltrating macrophages, respectively) and colored by pseudotime to show the changes in gene expression/phenotypic plasticity. (**B**) Feature plots of *Marco* versus informative genes to show the distribution of subpopulations with respect to pseudotime and origins of *Marco* induction with CCl_4_-induced fibrosis. (**C**) IPA using the magnitude of Moran’s test statistic (Table 1), not differential gene expression, for significantly induced genes along the pseudotime trajectory to identify associated pathways. Pathway analysis statistics were calculated by IPA using a right-tailed Fisher’s exact test to calculate *P* value.

To better understand the origin and gene programs associated with *Marco* expression, we reprocessed the CCl_4_ dataset for pseudotime trajectory analysis with the Monocle 3 package. Pseudotime trajectory analysis compares the similarity and incremental changes in gene expression between cells in a cluster, as well as the trajectory of the gain or loss of specific genes. This is important for showing the phenotypic plasticity of macrophages in response to injury. The phenotypic plasticity of resident (Node 1, *Clec4f*^+^) versus infiltrating (Node 2, *Itgam*^+^) macrophage populations was analyzed with a starting node in each population and convergence/divergence of specific subpopulations (Figure 3A). Feature plots highlight the distribution of *Clec4f*-expressing Kupffer cells versus *Itgam*-expressing infiltrating macrophages and the induction and distribution of *Marco* versus damage-associated *Trem2* (28, 29) expression along monocle3_pseudotime in (Figure 3B). Indeed, without sorting or supervised analysis, spatial autocorrelation analysis (i.e., Moran’s *I* values) quantitatively showed that *Marco* was one of the most strongly and significantly induced genes across pseudotime and highlighted that *Marco*^+^ cells emerged from the resident Kupffer cells, infiltrating macrophage subpopulations, and the convergence of the “Kupfferized” infiltrating cell-derived Kupffer cells (Table 1). Because other notable functional genes, including pathogen receptors and matrix metalloproteinases (MMPs), were apparent in the spatial autocorrelation analysis, the full list and magnitude of Moran’s test statistic values (not differential gene expression fold changes) were then subjected to IPA to identify the associated gene programs/pathways that were induced across pseudotime (Figure 3C and Table 1). Many pathways were associated with immune cell activation, but those associated with cell-cell interaction and phagocytosis were also significantly increased. Thus, pseudotime analysis allowed us to examine macrophage plasticity in the scope of CCl_4_ administration, demonstrating that *Marco*^+^ macrophages do not exclusively arise from *Clec4f^+^Itgam^–^* embryonic-derived Kupffer cells, but a small population also arises from infiltrating macrophages and infiltrating macrophage–derived Kupfferized cells, and they capture induction of important functional pathways. Our data support the fact that not all *Marco*^+^ macrophages are equivalent. The background cell of origin and microenvironment-related polarization program may strongly influence the overall phenotype of specific *Marco^+^* macrophage populations and highlights the importance of understanding macrophage heterogeneity. *Marco* expression increased apparent phagocytic capacity at baseline, and CCl_4_ administration increased the *Marco*^+^ cell population. Despite this, fibrosis still developed and was associated with downregulation of phagocytosis pathways in *Marco*^+^ macrophages from the CCl_4_ group compared with olive oil. This compelled us to characterize the properties of specific subpopulations of *Marco^+^* macrophages.

To further analyze the phenotype and origin of MARCO^+^ macrophages induced during liver fibrosis, we did mass cytometry (CyTOF) using a panel of antibodies (see Supplemental Methods) focused on macrophage markers. The results obtained show a shift in immune cell populations when comparing olive oil versus CCl_4_-induced fibrosis (Supplemental Figure 5A). The analysis returned 28 cell clusters, with the majority of them corresponding to macrophages (Supplemental Figure 5, B and C). MARCO expression levels can be observed in areas corresponding to cluster 19, which correspond to Kupffer cells identified by the expression of CLEC4f (Supplemental Figure 5D), but not exclusively, as higher MARCO levels can be observed scattered across other clusters (2, 7, 8, 18, 19, 23). Next, we subset MARCO^hi^ cells, and analyzed the expression of infiltration markers CD11b and CX3CR1. We found that a population of MARCO^hi^ cells, double positive for these 2 protein markers (cluster 7 and 8), is induced with fibrosis, which provides protein-level validation of the transcriptomic results, showing infiltrating MARCO^+^ macrophages (and MARCO^+^ infiltrating macrophage–derived Kupffer cells) in the liver (Supplemental Figure 5E).

Going forward, we did not always specifically attempt to discriminate between Kupffer cell subtypes and non-Kupffer cell macrophages due to assay limitations. Both populations increased *Marco* in response to injury, and the data further indicate that the majority of the infiltrating *Marco*^+^ cells had adopted the Kupffer cell phenotype *Itgam^+^Clec4f^+^*. Since Kupffer cells are macrophages, but not all macrophages are Kupffer cells, the term macrophage is used for all of these populations unless otherwise specified for a specific subset.

### MARCO expression is enhanced in areas with low collagen deposition in chronic liver fibrosis.

**Figure 4:**
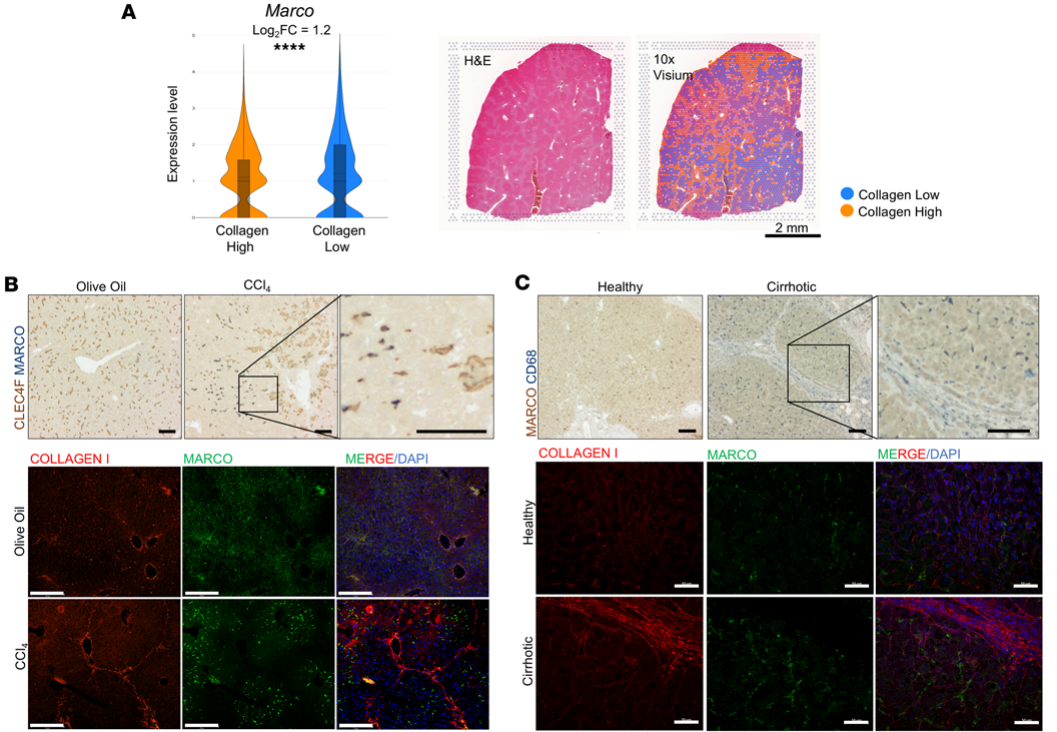
MARCO^+^ macrophages are increased in chronic liver fibrosis but localize to nonfibrotic regions. (**A**) 10X Visium CCl_4_ mouse liver sample demonstrating the colocalization of lighter fibrotic regions visible with H&E staining versus the designated collagen high and low “spots” used for differential expression of *Marc*o with violin plot relative to collagen type I in CCl_4_-induced fibrotic mouse liver. Collagen high represents fibrotic zones, collagen low represents nonfibrotic zones. Log_2_FC = 1.2; ****P < 0.0001 in **A**. (**B**) Representative images of CLEC4F macrophage marker and MARCO immunostaining as well as collagen type I and MARCO immunostaining in liver tissue sections of mice after chronic administration of olive oil or CCl_4_. The homogeneous distribution of macrophages in the olive oil group differs from the chronic fibrosis group by an increased macrophage population that segregates by MARCO^+^ cells that specifically do not colocalize with fibrotic collagen versus MARCO^–^ populations that have a distribution similar to the collagen staining. (**C**) Representative images of CD68 macrophage marker and MARCO immunostaining by IHC as well as immunofluorescence to show that the same distribution of MARCO^+^ macrophages compared with regions of fibrosis is seen in liver tissue sections from patients with and without cirrhosis as in the olive oil versus CCl_4_ fibrotic mouse model. IHC scale bar: 50 μm; IF scale bar: 275 μm. Each image is representative of 3 patient-derived or animal-derived samples.

The homogeneous, sinusoidal distribution of macrophages observed in healthy liver tissue was perturbed with chronic fibrotic injury in CCl_4_ mice, as well as human cirrhotic liver. Specifically, macrophages increased overall and appeared to concentrate in areas of fibrosis (Supplemental Figure 6). However, the behavior of specific macrophage populations, especially *Marco*^+^ macrophages was unknown. To understand the spatial distribution of *Marco*^+^ macrophages during liver fibrosis, we first analyzed our previously described spatial transcriptomics dataset (Gene Expression Omnibus [GEO], GSE259363) (30). Based on collagen type I expression, the liver was subset into fibrotic versus nonfibrotic zones (30). *Marco* was expressed primarily in the nonfibrotic zones with low collagen expression (Figure 4A). This finding was confirmed via IHC and immunofluorescence staining of MARCO^+^ macrophages in mouse and human liver tissue sections (Figure 4, B and C). MARCO^+^ cells were localized primarily to nonfibrotic areas of the liver from CCl_4_ group mice, indicating the macrophages localized to the fibrotic septa were MARCO^–^. Indeed, the fibrotic septa contained primarily MARCO^–^CLEC4F^+^ macrophages, with minimal numbers of double-positive macrophages in this region (Figure 4B). This spatial distribution pattern was also evident with MARCO^–^CD68^+^ macrophages in human cirrhotic tissue samples (Figure 4C). Taken together, these results suggest that MARCO^+^ macrophages are increased and tend to accumulate in the nonfibrotic areas in the liver during chronic fibrosis.

The aforementioned results led to the question of whether the MARCO^+^ macrophages have reduced migratory capacity toward the areas of fibrosis. To answer this question, *Marco* was transfected into RAW 264.7 mouse macrophage cells, which do not endogenously express *Marco*. We then analyzed migration in response to CXCL9, which is a key chemokine regulating the redistribution of macrophages in the liver (31). *Marco* overexpression significantly reduced migration at baseline and chemotaxis in response to CXCL9 stimulation (Supplemental Figure 7A). Phalloidin staining uncovered cytoskeletal remodeling, also potentially affecting migration capacity (Supplemental Figure 7B). Concordant with the migration results, we found that MARCO^+^ macrophages also express lower levels of *Cxcr3*, the receptor for CXCL9, compared with MARCO^–^ macrophages by qPCR (Supplemental Figure 7C). Thus, our results demonstrate that macrophage populations are not only increased in response to fibrotic injury overall but are spatially segregated based on MARCO expression, with regions of fibrosis/increased collagen I expression associated with MARCO^–^ cells and MARCO^+^ concentrated in the uninjured parenchyma. Furthermore, the specific localization of MARCO^+^ macrophages in nonfibrotic areas can be explained by a reduction in functional pathways associated with migration as evidenced by lack of response to CXCL9-induced chemotaxis, and low *Cxcr3* expression.

### MARCO^+^ macrophages have antiinflammatory, antifibrotic, and prophagocytic characteristics.

**Figure 5:**
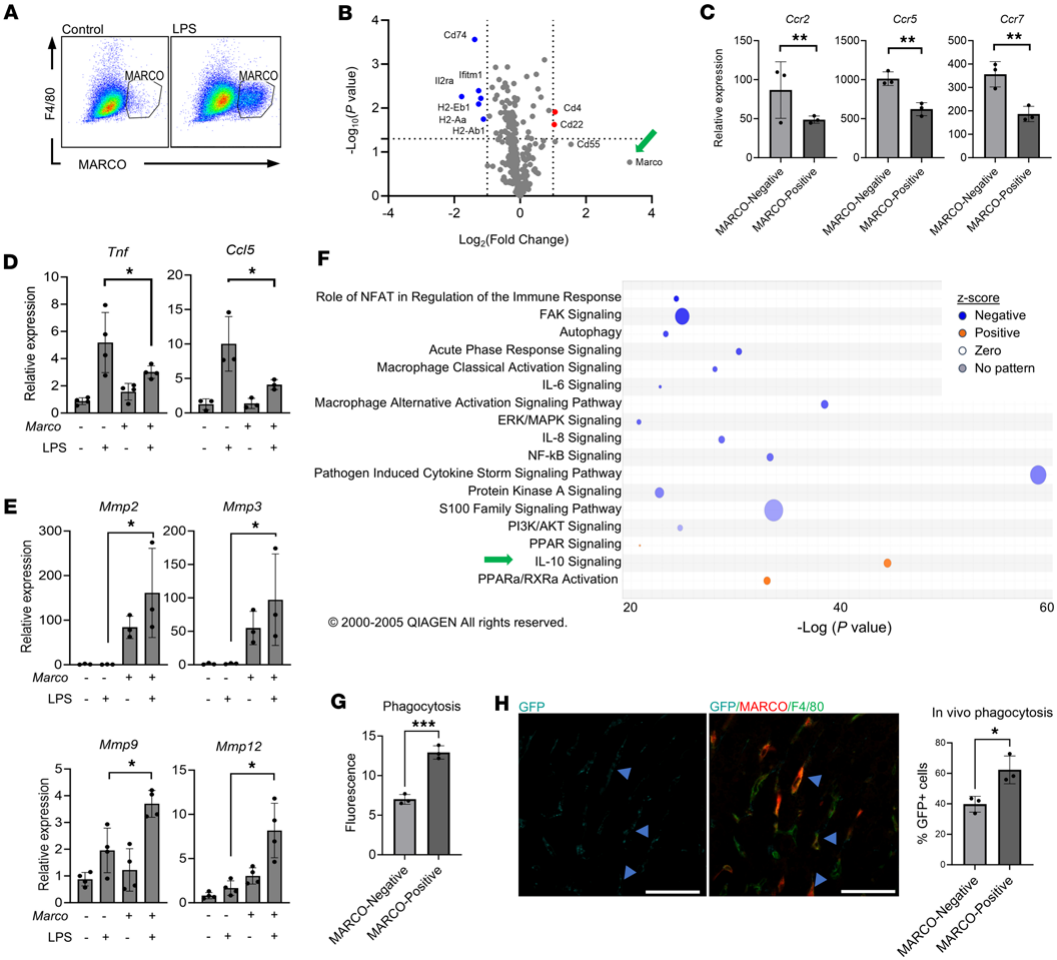
LPS induces a MARCO^+^ population in BMDMs with antiinflammatory characteristics. (**A**) Flow cytometric analysis of F4/80 and MARCO double-positive BMDMs before (left panel) and after (right panel) treatment with LPS. (**B**) Gene expression analysis of immune-related genes in BMDMs stimulated with LPS. Volcano plot shows differentially expressed genes, upregulated (red) or downregulated (blue), in MARCO^+^ versus MARCO^–^ BMDMs. (**C**) Expression of cytokine receptors *Ccr*2, *Ccr*5, and *Ccr*7 (in MARCO^–^ and MARCO^+^ BMDMs). (**D**) Expression of proinflammatory cytokines *Tnf* and *Ccl5* in MARCO^–^ and MARCO-overexpressing Raw 264.7 macrophages with and without LPS stimulation. (**E**) Expression of matrix metalloproteinases (MMPs) *Mmp2*, *Mmp3*, *Mmp9*, and *Mmp12* in MARCO^–^ and MARCO-overexpressing Raw 264.7 macrophages with and without LPS stimulation. (**F**) IPA of the nCounter fibrosis panel in MARCO^+^ BMDMs shows increased antiinflammatory IL-10 signaling and decreased inflammatory pathways. (**G**) Phagocytic capacity assay of MARCO^–^ and MARCO^+^ BMDMs, measured by internalization of fluorescent latex beads. (**H**) In vivo phagocytosis assay in CCl_4_ mice using GFP-labeled *E.coli* (arrowheads) and MARCO and GFP costaining, demonstrating increased phagocytic capacity in MARCO^+^ versus MARCO^–^ macrophages as seen in vitro in panel. Each image is representative of 3 mouse samples. (**G**). Scale bar: 50 μm. ***P < 0.001; **P < 0.01; *P < 0.05. IPA (Qiagen) *P* values were calculated via a right-tailed Fisher’s exact test. nCounter results were analyzed using nSolver software with significance determined by heteroscedastic *t* test (Welch’s). All other results were analyzed using 2-way ANOVA and Tukey’s comparisons test. Each experiment was conducted at least 3 times.

Since migration capacity alone is not sufficient to explain the phenotype of MARCO^+^ macrophages, we analyzed other functional characteristics using transcriptomics. The availability (Supplemental Figure 8A) and tolerance to manipulation of Raw264.7 cells and BMDM are substantially better than tissue-resident macrophages, and the BMDM potentially translate more readily to future therapeutic applications (32). Therefore, we used MARCO^+^ BMDM or *Marco*-overexpressing Raw264.7 for in vitro validation of the previous in silico analysis as well as additional transcriptomic studies. BMDMs were obtained by isolating mouse bone marrow hematopoietic progenitors and differentiating them with macrophage CSF treatment for 7 days. *Marco* is expressed at exceptionally low levels in WT BMDMs; therefore, we induced *Marco* expression by treating BMDMs with LPS using PBS as a vehicle (33). Only 2% of the vehicle-group F4/80^+^ population of BMDMs were positive for MARCO; however, LPS stimulus increased the percentage of MARCO^+^ cells in this population to 25% (Figure 5A). We then sorted F4/80^+^MARCO^–^ and F4/80^+^MARCO^+^ subpopulations with FACS and confirmed *Marco* mRNA and MARCO protein levels in each subpopulation with immunoblot and qPCR analysis, respectively (Supplemental Figure 8, B and C). Upstream regulator analysis of endogenous Marco^+^ macrophages further demonstrate the physiologic relevance of LPS induction of *Marco* (Supplemental Figure 9).

To assess the proinflammatory potential of MARCO^–^ and MARCO^+^ macrophages, we performed gene expression analysis of the sorted BMDM subpopulations with the NanoString nCounter, Fibrosis, and Immunology panels. The global differential gene expression from the total NanoString nCounter array immunology probe set for MARCO^+^ versus MARCO^–^ BMDMs is shown in a volcano plot (Figure 5B). From immunology panel, we found that expression levels of the migration-related genes *Ccr2*, *Ccr5*, and *Ccr7* were lower in MARCO^+^ BMDMs than in MARCO^–^ BMDMs (Figure 5C). We also analyzed sorted BMDMs after MARCO induction with nCounter fibrosis panel. MARCO^+^ BMDMs showed lower expression of the proinflammatory mediator and higher levels of several MMPs (*n* = 2, data not shown). We validated these results using Raw 264.7 macrophage cell line where we found that over expression of MARCO is associated with lower levels of Tumor necrosis factor (*Tnf)* and Chemokine C-C motif ligand 5 (*Ccl5*) at basal conditions and after inflammatory stimulation with LPS (Figure 5D). We also detected higher levels of the collagen-degrading MMPs *Mmp2*, *Mmp3*, *Mmp9*, and *Mmp12* (Figure 5E). IPA of the transcription profiles from BMDM (Figure 5F) showed IL-10 signaling and similar upregulated pathways in MARCO^+^ compared with MARCO^–^ BMDMs, which is associated with an antiinflammatory phenotype.

Finally, to validate the tissue macrophage IPA results and confirm a potentially fundamental property of tissue restorative macrophages, we examined the phagocytic activity of MARCO^+^ versus MARCO^–^ BMDMs in vitro by measuring the uptake of fluorophore-labeled opsonized latex particles. Phagocytic activity was significantly higher in MARCO^+^ BMDMs than in MARCO^–^ BMDMs (FC = 1.72, *P* < 0.05) (Figure 5G). We further validated this finding by measuring phagocytosis in vivo using GFP-labeled *E*. *coli* injected into the portal vein of CCl_4_ group mouse livers. Costaining of MARCO, F4/80, and GFP revealed approximately 40% of MARCO^–^ cells were GFP^+^ while a significantly larger population of MARCO^+^ macrophages phagocytosed the GFP^+^ *E*. *coli* (Figure 5H). These results indicate a less inflammatory and highly phagocytic phenotype in MARCO^+^ BMDMs and intrahepatic macrophages, indicating possible antifibrotic or prorestorative characteristics.

These results are also notable for the differences between the MARCO^+^ BMDMs and the endogenous *Marco^+^* macrophage from the CCl_4_ injury model. Indeed, *Marco*^+^ macrophages were shown to have differences in phagosome formation by IPA when comparing *Marco*^+^ macrophages in the healthy olive oil control versus the CCl_4_ chronic fibrosis model (Supplemental Figure 3). This suggests that the microenvironment in which the *Marco*^+^ macrophages are derived could affect their function, such that the BMDMs may be more efficacious than endogenous macrophages in attenuating chronic fibrosis.

### Adoptive transfer of MARCO^+^ macrophages attenuates fibrosis in vivo.

**Figure 6:**
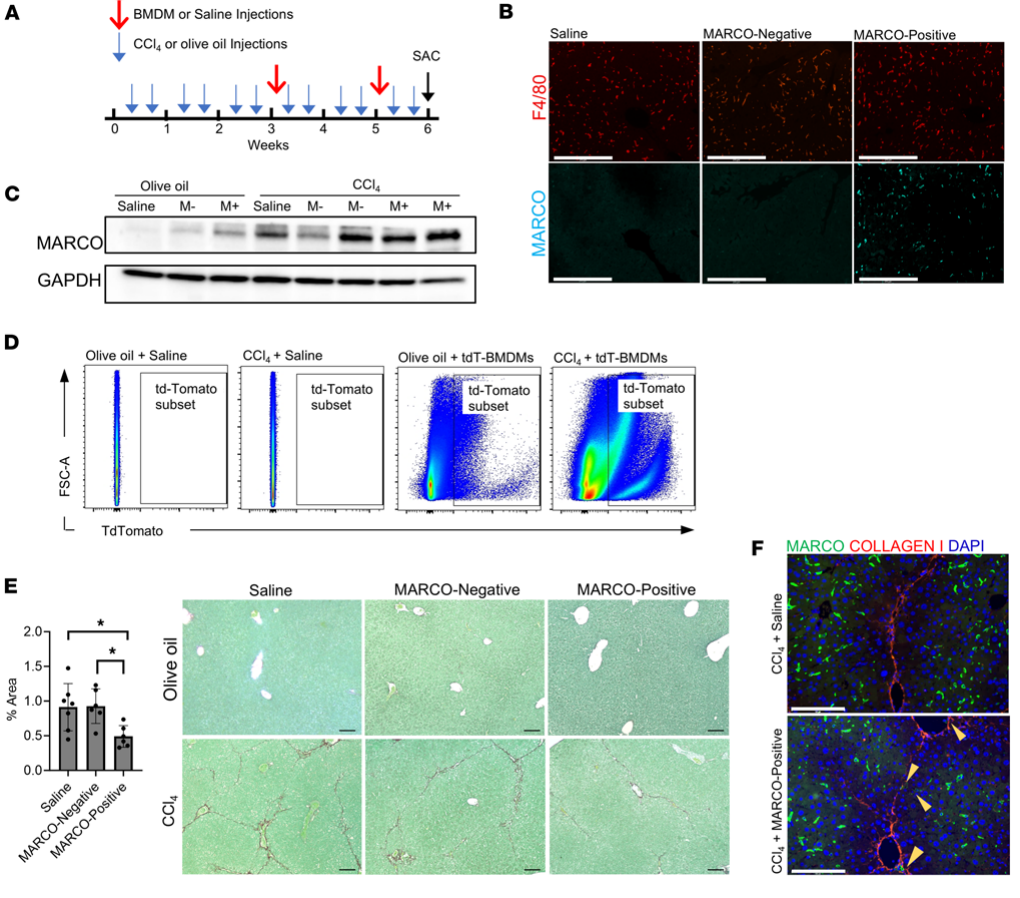
Adoptive transfer of MARCO^+^ BMDMs reduces fibrosis in vivo. (**A**) Schematic showing adoptive transfer protocol and CCl_4_ administration. Mice received 2 tail vein injections of 1 × 10^6^ MARCO^+^, MARCO^–^ BMDMs, or saline at 3 and 5 weeks of olive oil/CCl_4_ administration protocol. (**B**) Immunofluorescence staining of F4/80 and MARCO in liver tissue sections to confirm the presence of transferred MARCO^+^ BMDMs. Olive oil–administered controls were used to prevent interference from CCl_4_-induced endogenous MARCO^+^ macrophage staining. Scale bar: 275 μm (**C**) Representative immunoblots of MARCO and GAPDH (loading control) proteins in liver tissue lysates after olive oil or CCl_4_, with (M-, Marco^–^; M+, MARCO^+^) or without (Saline) macrophage adoptive transfer. (**D**) Analysis by flow cytometry of isolated liver macrophages from mice after receiving adoptive transfer of BMDMs that constitutively express tandem dimer Tomato fluorescent protein (tdT) or WT BMDM. (**E**) Picrosirius/fast green staining of collagen (i.e., fibrosis) in liver tissue sections from mice that received adoptive cell transfer. Quantification of picrosirius staining in CCl_4_-induced fibrotic livers is shown in the bar graph. Scale bar: 100 μm. (**F**) Representative immunofluorescence image of collagen type I and MARCO staining in fibrotic liver tissue sections from mice that received adoptive cell transfer. Scale bar: 100 μm. *P < 0.05. Statistical analysis was done using 2-way ANOVA and Tukey’s comparisons test. The cell transfer experiment was conducted on at least 6 animals per group.

Based on the reparative properties of *Marco*^+^ macrophages observed in vitro, we hypothesized that MARCO^+^ macrophages may improve liver fibrosis in vivo. To test this hypothesis, we conducted an allogeneic adoptive transfer experiment in which sorted MARCO^+^ and MARCO^–^ BMDM subpopulations (Figure 5A) were adoptively transferred into recipient mice during the CCl_4_ administration protocol (Figure 6A). To confirm the homing of exogenous macrophages to the liver, we used healthy mice with and without cell transfer to detect the presence of MARCO^+^ cells in the liver. Basal numbers of MARCO^+^ macrophages are exceptionally low in healthy livers (Figure 2F, Supplemental Figure 2, and Supplemental Figure 8A), so we could be confident that the increase in MARCO^+^ cells detected after adoptive transfer was due to the MARCO^+^ BMDMs and not endogenous cells. As a control, we also transferred MARCO^–^ BMDMs to a second group of control mice and observed no increase in MARCO staining compared with healthy liver. Thus, the BMDMs did not alter their MARCO expression during the transfer experiment. The number of total macrophages showed minor variation among the 3 conditions, as shown by staining F4/80 (Figure 6B). This finding was further confirmed by immunoblot analysis on whole livers. Notably, livers of mice from the olive oil group had higher levels of MARCO protein after transfer of MARCO^+^ BMDMs than saline or MARCO^–^ BMDM recipients. Still, they had lower MARCO protein levels than recipient mice with CCl_4_ (Figure 6C). These findings confirm that the exogenous MARCO^+^ BMDMs accumulated in the livers of recipient mice, and the accumulation was increased during CCl_4_-induced fibrosis. To further confirm that the increased accumulation of MARCO^+^ cells was due to exogenous MARCO^+^ macrophages integrating into the liver, we performed flow cytometry analysis utilizing fluorescent LPS-stimulated BMDMs isolated from tandem dimer Tomato–expressing (tdTomato-expressing) mice (tdT-BMDMs). Mice from olive oil or CCl_4_ groups were injected with tdT-BMDMs at weeks 3 and 5 of the CCl_4_ administration protocol, as described in Figure 6A. Flow cytometric analysis of isolated intrahepatic leukocytes showed tdTomato fluorescence in a cell population from tdTomato-BMDM recipient mice but not from the saline mice (Figure 6D). The tdTomato^+^ population increased from 1.52% in mice from the olive oil group to 26.8% in mice from the CCl_4_ group, indicating that the transferred BMDMs specifically homed to the injured liver (Figure 6D). Next, we investigated the effect of exogenous transferred MARCO^+^ macrophages on liver fibrosis. Picrosirius red staining revealed a significant decrease in collagen deposition in liver sections from recipients of MARCO^+^ BMDMs compared with saline or MARCO^–^ BMDM recipients, indicating that the CCl_4_-induced fibrotic phenotype was attenuated after adoptive cell transfer of MARCO^+^ BMDMs (*P* < 0.05) (Figure 6E). To confirm the effect of exogenous MARCO^+^ BMDMs in reducing the fibrosis in the CCl_4_ model, we used clodronate to deplete endogenous macrophages before the cell transfer of BMDM, which also express GFP for tracking of transferred cells. With this model, we validated our results, as we showed a reduction of collagen deposition in mice that received MARCO^+^ BMDMs compared with those that received MARCO^–^ BMDMs using picrosirius red staining and hydroxyproline level quantification (Supplemental Figure 10, A–C).

Our staining and spatial transcriptomics data show that endogenous *Marco*^+^ macrophages predominantly reside in the nonfibrotic areas of the liver after CCl_4_-induced fibrosis. Thus, we assessed potential changes in the spatial distribution of MARCO^+^ macrophages after transfer of MARCO^+^ BMDMs. In the livers of CCl_4_ group mice, MARCO staining was colocalized with collagen type I staining (Figure 6F), showing that the transferred MARCO^+^ BMDMs localized to the fibrotic zones of the injured liver. These observations demonstrate that MARCO^+^ BMDMs can successfully home to the injured liver following adoptive transfer and reduce collagen deposition and fibrosis.

### Adoptive transfer of MARCO^+^ macrophages downregulates ECM genes from hepatic stellate cells.

**Figure 7:**
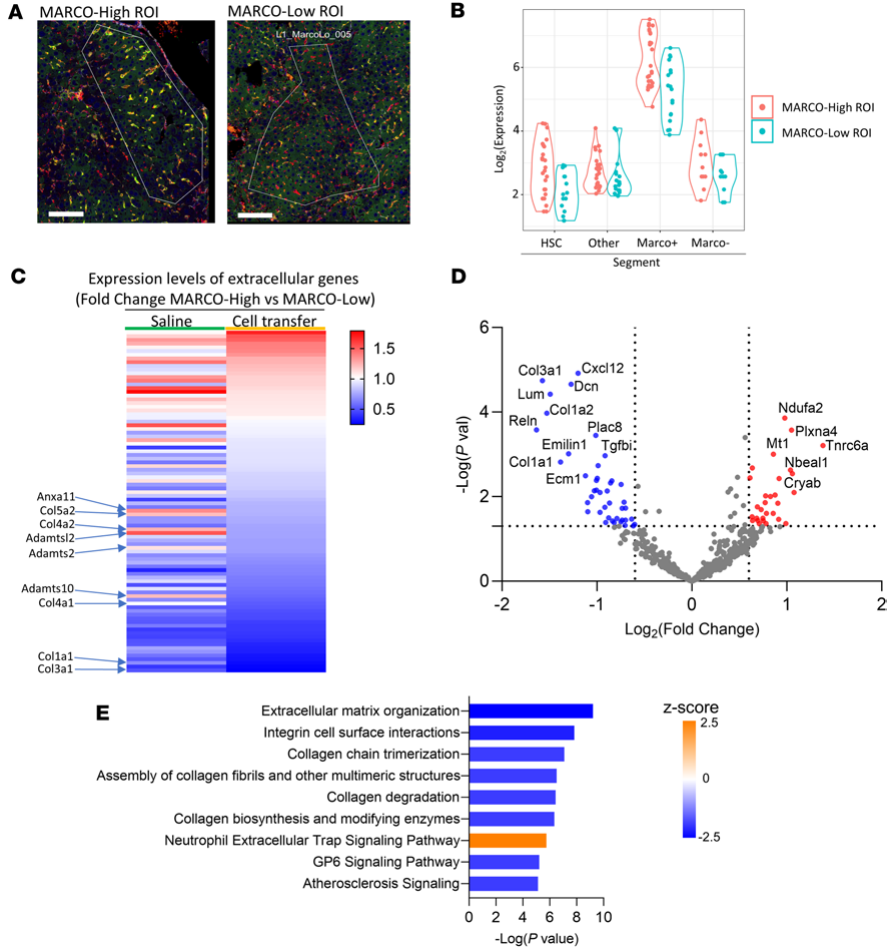
Adoptive transfer of *Marco*^+^ macrophages in vivo induces phenotypic changes in HSCs. (**A**) Regions of interest (ROI) for GeoMx DSP were selected based on IBA1^+^ and MARCO^+^ immunofluorescence staining. MARCO^Hi^ ROIs are MARCO^+^IBA1^+^ (yellow), and MARCO^Lo^ ROIs are MARCO^–^IBA1^+^ (red). Scale bar: 125 μm. (**B**) *Marco* transcript levels across all of the segments and ROIs for quality control. (**C**) Heatmap shows Extracellular gene expression fold change for HSC in *Marco*^hi^ versus *Marco*^lo^ ROIs. The first column shows CCl_4_-treated mice that did not receive cell transfer, and the second column shows CCl_4_-treated mice that received cell transfer. Rows were not clustered for this 2 column comparison, and the color gradient indicates the magnitude of fold change for Marco^Hi^ versus Marco^lo^ across all analyzed genes. Extracellular genes highlighted in the saline group are reduced in *Marco*^hi^ ROI with cell transfer. Extracellular gene list obtained from Gene Ontology consortium (www.geneontology.org). (**D**) Volcano plot of differentially expressed genes for DESMIN^+^ segments from MARCO^Hi^ compared with MARCO^Lo^ ROI after treatment with MARCO^+^ BMDMs. (**E**) IPA showing reductions in ECM-related pathways in DESMIN^+^ segments after MARCO^+^ BMDM cell transfer treatment. Scale bar: 125 μm. *P* values were calculated by IPA using a right-tailed Fisher’s exact test.

We used spatial transcriptomics to gain potential mechanistic insight into how MARCO^+^ macrophages reduce liver fibrosis in vivo. We analyzed the effect of MARCO^+^ macrophages on hepatic stellate cells (HSCs), the primary matrix-producing cells and key drivers of fibrosis, in fibrotic mouse livers after chronic CCl_4_ administration. The NanoString GeoMx Digital Spatial Profiler platform was used to obtain the transcription profile of HSCs colocalized with MARCO^+^ versus MARCO^–^ macrophages, in samples from mice that received CCl_4_+Saline compared with samples from mice that received CCl_4_+MARCO^+^ BMDMs. The goal was to determine if the MARCO^+^ BMDMs affected the fibrogenic capacity of the HSCs. To analyze the tissue, we labeled HSC using an anti-Desmin antibody (34), and macrophages were labeled using anti-IBA1 (panmacrophage marker) (35) and anti-MARCO. We selected regions of interest (ROIs) enriched in MARCO^+^ macrophages. These ROIs were subdivided into segments corresponding to HSCs, MARCO^+^ macrophages, and MARCO^–^ macrophages (Figure 7A), and each segment was individually sequenced, giving the transcription profile for the particular cell type (Supplemental Figure 11A). There was also a fourth segment denominated “Other,” which corresponded to everything else in the ROIs that was not HSC or Macrophage. The segment “Other” was used mainly as a segmentation control. As expected, MARCO^+^ segments within MARCO^hi^ ROI show the highest levels of *Marco* transcript compared with MARCO^Lo^ ROI. Detection of *Marco* transcript was minimal across the DESMIN and “Other” segments, indicating that the segmentation and collection of high-quality transcriptomic libraries was satisfactory (Figure 7B).

We then analyzed the transcriptomic profile of HSCs from MARCO^hi^ and MARCO^lo^ ROIs between the CCl_4_+Saline and CCl_4_+MARCO^+^ BMDMs groups. Again, the primary objective was to determine the effect of adoptive transfer of MARCO^+^ BMDMs on fibrosis. However, the secondary objective was to address the differences noted between the endogenous (injury-induced) tissue MARCO^+^ macrophages present in the CCl_4_ group (Figure 2 and Supplemental Figure 3) and the transferred MARCO^+^ BMDMs (Figures 5 and 6), which had been previously complicated by the fact that the cells were originally assessed using different transcriptomic platforms (10x scRNA-seq versus Nanostring NCounter). Specifically, the MARCO^Hi^ ROIs from the CCl_4_+Saline mouse liver sample correspond to the endogenous (injury-induced) MARCO^+^ macrophages characterized in Figure 2, while the MARCO^Hi^ ROIs from the CCl_4_+MARCO^+^ BMDMs sample correspond to a mixture of endogenous, as well as the BMDMs characterized in Figures 5 and 6. To visualize the transcriptomic effect of endogenous versus transferred MARCO^+^ macrophages during the development of fibrosis, we used a list of extracellular genes from the Gene Ontology Consortium to compare changes in HSC gene expression with the most important changes evident after MARCO^+^ BMDM adoptive cell transfer that include downregulation of several collagens and other ECM-component genes. (Figure 7C). To optimize the detection and analysis of HSC-specific genes, we subset the NanoString GeoMx dataset to 760 genes according to the strategy described in Supplemental Figure 11B. We started with the HSC conserved gene list from our previously published scRNA-seq dataset (36) and removed ambient/contaminant genes by selecting conserved genes expressed in more than 30% of cells in the HSC cluster and in fewer than 15% of non-HSCs. (Supplemental Figure 11B). Differential gene expression analysis of HSCs from DESMIN^+^MARCO^Hi^ versus DESMIN^+^MARCO^Lo^ segments indicated that there was a downregulation of ECM-related genes, including structural (collagens) and modulatory (*Dcn*, *Ecm1*) genes, after administration of MARCO^+^ BMDMs in CCl_4_-induced fibrosis (Figure 7D). This significant downregulation was not observed in the CCl_4_+Saline group livers, which only contained the endogenous (injury-induced) MARCO^+^ macrophages and did not receive any additional MARCO^+^ BMDMs (Supplemental Figure 11C). IPA of the differential gene expression shown in Figure 7D demonstrated that the most significant downregulated HSC pathways affected by MARCO^+^ macrophage adoptive cell transfer correspond to ECM organization, collagen degradation, biosynthesis, polymerization, and fibril assembly (Figure 7E). The significant downregulation of ECM genes was not observed when comparing HSC in MARCO^hi^ versus MARCO^lo^ areas without adoptive transfer of macrophages (Supplemental Figure 11D). These results indicate that MARCO^+^ BMDM adoptive transfer reduces fibrosis by directly downregulating ECM pathways in HSC.

## Discussion

Despite the high worldwide burden of chronic liver diseases, the lack of efficient therapy persists. Recently, macrophage-based therapies have been considered for the treatment of chronic conditions (37, 38). Due to the high phenotypic plasticity of macrophages, emergent high-throughput technologies, including scRNA-seq and spatial transcriptomics, improve the study of macrophage heterogeneity and its therapeutic potential (8, 39–45). For example, a subpopulation of recruited macrophages has been shown to transdifferentiate into Kupffer cells in the fatty liver (42), and several Kupffer cell subpopulations are known to maintain liver function in alcohol-mediated liver disease (43). However, less is known about macrophage heterogeneity during fibrosis. In this study, we sought to understand the importance and potential benefits of specific macrophage subpopulations within the liver fibrotic niche. We observed that chronic fibrosis not only resulted in an increase in total macrophage number and transcriptional heterogeneity but also induced a macrophage subset that was characterized by significant upregulation of *Marco*, spatial relocalization, and a potential tissue restoration phenotype. MARCO^+^ macrophages were sparse and homogeneously dispersed across the sinusoids in healthy human and mouse liver tissue. However, in the context of fibrosis, these cells expanded and relocalized to form concentrated niches in nonfibrotic areas. The endogenous MARCO^+^ macrophages were all but absent in fibrotic areas, which were dominated by CD68^+^MARCO^–^ macrophages. This spatial redistribution raised the question of whether MARCO^+^ macrophages possess antifibrotic properties that maintain their niche fibrosis-free or if they are unresponsive to chemotactic signals derived from fibrotic areas. Our main findings using MARCO^+^ BMDMs indicate that it could be a mix of both mechanisms. Notably, we also demonstrated that the adoptive transfer of MARCO^+^ BMDMs was sufficient to reduce CCl_4_ fibrosis in vivo, which opens the possibility of macrophage-based fibrosis treatments in the future.

While MARCO^+^ macrophages increased with CCl_4_-induced fibrosis in our study, they were not colocalized with fibrotic regions. This result could be explained by the lack of migratory response of MARCO^+^ macrophages to CXCL9, an important mediator of macrophage migration in the liver (31), and could be supported by low expression of *Cxcr3*, which encodes the CXCL9 receptor. In addition to the changes in migratory behavior, we sought to understand if the lack of fibrosis surrounding MARCO^+^ macrophages was due to a prorestorative phenotype. A recent study by Miyamoto et al. (40) reported that macrophages with elevated levels of MARCO might protect against inflammation caused by gut bacteria. However, the role of this MARCO^+^ subpopulation of macrophages during fibrosis was not clear. Notably, our transcriptomic analysis of tissue resident/infiltrating macrophage–derived Kupffer cells and *Marco*^+^ BMDM revealed potential phenotypic differences between *Marco^+^* macrophages based on the microenvironment in which they were induced, which may also explain the inconsistent results reported in other studies about the nature of MARCO^+^ macrophages (46–49). For example, IPA revealed the downregulation of functional pathways, such as phagocytosis in *Marco*^+^ macrophages derived from CCl_4_ compared with olive oil *Marco*^+^. However, MARCO^+^ BMDM demonstrated high phagocytic activity. Phagocytosis contributes to tissue restoration by participating in ECM remodeling and fibrosis reversal. During ECM remodeling, proteases like MMPs degrade collagen and other ECM components, followed by debris removal by phagocytic cells. (50, 51). For a deeper understanding of MARCO^+^ macrophages/Kupffer cells in situ, we will need specific and targeted future analysis. Nonetheless, rather than assume that the *Marco*^+^ BMDMs would be incapable of reaching injured regions, the data suggesting a potentially prorestorative phenotype prompted us to assess whether bone marrow–derived MARCO^+^ macrophages could be used to home to fibrotic niches in the injured liver to reverse CCl_4_-induced fibrosis. Indeed, our data indicate that the adoptive transfer of MARCO^+^ BMDMs successfully reduced fibrosis (i.e., collagen deposition) in the livers of CCl_4_-induced fibrotic mice, supporting the potential of MARCO^+^ macrophages for cell-based therapies to treat chronic liver disease.

Adoptive transfer of MARCO^+^ macrophages presents a compelling approach for liver fibrosis, as demonstrated in our CCl_4_-induced chronic fibrosis mouse model. Liver fibrosis results from the persistent activation of HSCs, which overexpress fibrogenesis genes and contribute to excessive ECM deposition. The ability of MARCO^+^ macrophages to suppress these genes, as evidenced by our NanoString GeoMx datasets, underscores their potential to reprogram the liver microenvironment and alleviate fibrosis. Specifically, the repression of key fibrogenesis genes, including *Col1a1*, *Col3a1*, *Dcn*, *Ecm1*, *Emilin*, *Reln*, and *Cxcl12*, highlights their multifaceted role in mitigating fibrosis and restoring liver function. Two of the most notable targets of MARCO^+^ macrophages are *Col1a1* and *Col3a1*, which encode the primary ECM components in fibrotic tissues (52). By reducing *Col1a1* and *Col3a1* expression, MARCO^+^ macrophages directly limit the accumulation of structural ECM proteins, mitigating the stiffening and scarring characteristics of advanced fibrosis. Similarly, their effect on *Dcn* (decorin), a small leucine-rich proteoglycan involved in ECM assembly and regulation (53), further supports their role in modulating ECM composition and preventing fibrotic progression. The suppression of *Ecm1* (ECM protein 1) and *Emilin* (elastin microfibril interface-located protein) by MARCO^+^ macrophages indicates their influence on ECM dynamics. ECM1 is known to regulate ECM organization and interaction with growth factors (54), while EMILIN modulates ECM elasticity (55). Their downregulation implies that MARCO^+^ macrophages not only reduce ECM deposition but also restore its mechanical properties, creating a microenvironment conducive to tissue repair. Collectively, these findings suggest that MARCO^+^ macrophages exert antifibrotic effects through both direct and indirect mechanisms. Their ability to modulate HSC activity, ECM composition, and inflammation reflects their important role in restoring liver homeostasis. However, several questions remain unanswered. For instance, the exact molecular pathways by which MARCO^+^ macrophages achieve these effects on HSCs are yet to be fully elucidated. Additionally, their long-term efficacy and integration within the liver microenvironment requires further investigation.

In summary, our study identifies subpopulations of liver macrophages that may be broadly categorized as profibrotic or antifibrotic based on *Marco* expression. Indeed, adoptive cell transfer with MARCO^+^ macrophages elicited an antifibrotic restorative phenotype in a mouse model of chronic liver fibrosis. Our approach offers a promising path for cell-based therapies capitalizing on the antifibrotic functions of macrophages to improve liver function.

## Methods

### Sex as a biological variable

Our study examined male and female animals, and similar findings are reported for both sexes.

### In vivo experiments

WT C57BL/6 male and female mice were purchased from Envigo (Envigo RMS LLC). Eight-week-old mice were i.p. administered 1 mL/g body weight carbon tetrachloride (CCl_4_) (319961; Sigma-Aldrich) or olive oil twice weekly for 6 weeks. For adoptive transfer experiments, MARCO^+^ and MARCO^–^ macrophages were obtained by fluorescence-activated cell sorting (FACS), as described below. Sorted cell populations were resuspended in normal saline (0.9% NaCl) at a concentration of 1x10^7^ cells/mL, and 0.1 mL of cell suspension was injected into the tail vein with a 29-gauge needle at 3 and 5 weeks of olive oil or CCl_4_ administration. Livers were evaluated with picrosirius red staining of tissue sections, immunoblotting, immunofluorescence staining, qPCR, scRNA-seq, and spatial transcriptomics. For the Clodronate-induced macrophage depletion assay, 100 μL of liposomes containing clodronate or empty liposomes (Standard Macrophage Depletion Kit, CLD-8901, Encapsula NanoSciences) were injected i.p. 2 days before each BMDM injection.

### In vivo phagocytosis assay

#### E.

*coli* bacteria (C3040H, New England Biolabs) were transformed using pGLO (1660405, Bio-Rad) plasmid for the expression of GFP protein. Single colonies were selected and cultured in Lysogenic Broth supplemented with 100 μg/mL of ampicillin to an optical density of 1 measured at 600 nm. GFP-expressing bacteria were washed with 1× PBS and concentrated in half of the original volume, and 50 μL of this cell suspension was used for each mouse. After 6 weeks of fibrosis induction with CCl_4_, the mice were anesthetized, and the bacterial suspension was injected i.v. into the portal vein. Then, the mice were kept on a heating pad for 30 minutes before euthanizing and harvesting the liver. Phagocytosis was measured by immunofluorescence staining of liver sections with GFP, MARCO, and F4/80 antibodies. At least 10 images per mouse were analyzed using ImageJ Software (NIH) (56) for colocalization masking and colocalized cells were manually counted. The results are shown as percentage of macrophages positive for GFP signals.

### Mouse bone marrow–derived macrophage isolation and culture

Mouse bone marrow–derived macrophages (BMDMs) were isolated from 8- to 12-week-old C57BL/6 WT (Envigo RMS LLC), tdTomato-expressing mice (JAX stock #007676), or GFP-expressing mice (JAX stock #004353). Mice were humanely euthanized by CO_2_ exposure. Long bones from the hind limbs were collected, and the epiphyses were removed. Bone marrow was obtained by flushing the bones with a syringe (23-gauge needle) filled with RPMI 1640 culture medium (22400-039; Gibco), 10% FBS (S11150H; R&D Systems), 1% penicillin/streptomycin (15140122; Gibco), and 0.2% amphotericin B (15290-018; Gibco). Bone marrow suspensions were filtered through a 70 mm mesh, and red blood cells were lysed with ACK lysis buffer (A1049201; Gibco). After washing, bone marrow cells were plated and maintained in RPMI 1640 (22400-039; Gibco) culture medium supplemented with 10% FBS (S11150H; R&D Systems), 1% penicillin/streptomycin (15140122; Gibco), 0.2% amphotericin B (15290-018; Gibco), and 25 ng/mL recombinant mouse macrophage colony-stimulating factor (M-CSF, 576406; BioLegend, Inc). After 7 days of culture, the cells were washed with 1× DPBS (14040133, GIBCO) to remove M-SCF and stimulated with 100 ng/mL LPS (tlrl-eblps; InvivoGen) in RPMI medium for 12 hours before FACS or functional assays.

### Fixed scRNA-seq

#### Sample preparation.

Mouse livers were harvested after 6 weeks of twice weekly carbon tetrachloride (CCl_4_) or olive oil administration. In situ tissue digestion was performed by perfusing the liver with collagenase P (COLLP-RO, Millipore Sigma) at a concentration of 64 mg/mL in a balanced salt solution. Digested livers were then homogenized in a petri dish in a solution containing 0.15 mg/mL DNase I (11284932001, MilliporeSigma). The intrahepatic leukocyte fraction was obtained by 25%–50% Percoll gradient (57). Cells were processed following the 10× Genomics protocol for cell/nuclei fixation (CG000782). Briefly, suspensions containing up to 10 million cells were spun down at 350 × *g* for 5 minutes at 4°C, before being resuspended in 1 mL of Fixation Buffer (4% formaldehyde, 1× Fix and Perm Buffer – 10x Genomics PN-2001301). Cells were fixed for 24 hours at 4°C. To halt fixation, cells were spun down at 850 × *g* for 5 minutes at room temperature and quenched with 0.5 mL of Quenching Buffer (1× Quench Buffer – 10x Genomics PN-2001300). Cell concentration and viability were assessed via AO/PI staining using a Cellometer K2 Cell Counter (Nexcelom), before being processed according to the 10x Genomics Flex protocol for hybridization.

#### Sample hybridization.

All hybridizations followed 10x Genomics recommendations (CG000787 Rev B). Hybridizations were set up in 40 μL of hybridization mix with 10 μL of Mouse WTA probes (10x Genomics PN-2001275-2001278). Hybridizations were performed at 42°C for 16–24 hours. Afterward, samples were diluted in Post-Hyb Wash Buffer and measured via AO/PI staining with a Cellometer K2 Cell Counter. Pooled hybridized cells were washed 3 times in Post-Hyb Wash Buffer for 10 minutes at 42°C . After washing, cells were resuspended in Post-Hyb Resuspension Buffer, filtered through a Miltenyi Biotec 30 μm filter, and measured to determine the volume required for the GEM-X Flex Gene Expression run. Before probe hybridization, an equal number of cells from each fixation were pooled (prehybridization normalization) to ensure equal contribution per sample.

#### Gene expression flex run.

For Gel Beads-in-emulsion (GEM) generation, we followed the 10x Genomics protocol and guidelines on cell and reagent volumes based on targeted cell recovery. We aimed for 20,000 cells per sample. For the probe mixing strategy experiment, we targeted 80,000 cells per pool with equal contribution from each sample, except for the differential pooling strategy, where contributions varied. After loading the GEM-X FX Chip on the Chromium X, GEMs were recovered and processed as per 10x Genomics instructions. The product was then preamplified and indexed for library construction. Libraries were quality-checked using the fragment analyzer.

#### Sequencing.

Libraries were sequenced on the Illumina Nova-Seq X 1.5B platform according to 10x Genomics recommended settings, with a minimum of 10,000 read pairs per cell. For experiments involving probe mixing strategies, sequencing was conducted at a depth of 10,000 read pairs per sample, yielding 80,000 cells per library.

### Bioinformatic analysis

Quality control parameters were set to optimize the recovery of high-quality, singlet nonparenchymal cells rather than hepatocytes (nFeature_RNA > 200 and nCount_RNA < 30000 and percent.mt < 10). Within Seurat (58), the sctransform (SCT), v2, package was used to normalize and inspect samples before integration and unsupervised reclustering at a resolution of 0.1 (59, 60). Cell clusters were identified according to established marker genes and the macrophage cluster subset for downstream analysis. Next, the “PrepSCTFindMarkers” function was run, followed by Seurat “FindMarkers” for case versus control differential gene expression for the macrophage cluster. The macrophages were then bioinformatically pooled and resorted for specific subpopulations of interest based on the experimental group and genes of interest. Comparisons included *Marco*^+^ versus *Marco*^–^ within cells from CCl_4_ group or *Marco*^+^ cells from CCl_4_ group mice compared with olive oil group, for example. “RecorrectUMI” was set to FALSE to account for the subsetting of the cells, and “FindMarkers” was used to calculate differential gene expression using the default Wilcoxon rank sum test and Benjamini-Hochberg–adjusted *P* values to control for multiple comparisons. Data were also analyzed through the use of Qiagen IPA (61).

### Pseudotime analysis with Monocle 3

Seurat (v5.1) objects for the olive oil and CCl_4_ groups were regenerated in R software, v4.3.2 (62), subset to nFeature_RNA > 200, nCount_RNA < 30,000, and percent.mt < 10 and normalized with NormalizeData rather than SCT in preparation for Monocle 3 software, v1.3.4 (63). The data were then scaled and subjected to principal component analysis (PCA) at 1:30 dimensions, dimension reduction via uniform manifold approximation and projection (UMAP), and unsupervised clustering (64). Seurat objects were interconverted to “as.cell_data_set” with SeuratWrappers, v0.3.2. The macrophages self-segregated into a single partition that was subset via the interactive “choose_cells” tool before reclustering, and the trajectory was calculated via “learn_graph.” Cells were ordered for pseudotime analysis via manual selection in “order_cells” and calculated by using 2 single-node starts, as well as a starting node in each of the 2 major clusters. Trajectory and pseudotime-colored plots were generated on the monocle objects and compared with FeaturePlots generated on the converted Seurat object. To determine which genes varied across pseudotime, Moran’s test statistic, Moran’s *I*, and *P* and *q* values were calculated with “graph_test” on the monocle object. The sorted gene lists were compared for the 3 node configurations and determined to be identical. Thus, the values for Moran’s *I* did not change according to the number or location of starting nodes. Consequently, the observed gene induction effect was not based on the choice of starting node in the pseudotime analysis. IPA was run on the sorted gene lists with the magnitude of Moran’s test statistic values used as the log (fold change). The threshold for significance was set to *P* < 0.05.

### NanoString nCounter expression analysis

Gene expression analysis was performed with the NanoString nCounter Analysis System (Bruker Spatial Biology Inc.) according to the manufacturer’s instructions. Briefly, total RNA was diluted to a concentration of 20 ng/mL, and 5 μL of diluted sample was hybridized with the nCounter Mouse Immunology Panel (XT-CSO-MIM1-12) or Mouse Fibrosis Panel (XT-CSO-MFIB2-12) for 18 hours at 65°C. Results were analyzed using the NanoString nCounter Profiler. Data analysis and quality assessment were performed with nSolver Analysis Software, v4.0 (Nanostring, Spatial Biology Inc.), with significance determined by heteroscedastic *t* test (Welch’s). All *t*-tests are 2-tailed, except when indicated (right-tailed for IPA).

### NanoString GeoMx digital spatial profiling

Formalin-fixed paraffin-embedded (FFPE) tissue sections of CCl_4_-administered livers were prepared according to the instructions in the Slide Preparation User Manual (MAN-10150-01, NanoString Technologies) using the GeoMx RNA Slide Prep Kit for FFPE (GMX-PREP-RNAFFPE-12, NanoString Technologies). Briefly, antigen retrieval was performed using 1× Tris-EDTA (pH 9.0) buffer, followed by Proteinase K digestion (0.1 mg/mL) and overnight in situ hybridization with the GeoMx Whole Transcriptome Atlas Mouse RNA Panel (GMX-RNA-NGSMsWTA-4, NanoString Technologies). Then, morphology marker staining was performed using antibodies against DESMIN (HSCs), MARCO, and IBA1 (macrophage), along with corresponding secondary antibodies (Supplemental Table 1). Immunofluorescence images were acquired with the GeoMx Spatial Digital Profiler instrument. Fourteen ROIs were selected manually and classified as MARCO^Hi^ and MARCO^Lo^ areas, using MARCO signal levels as a reference. ROI segmentation was automatically performed by the GeoMx DSP analysis suite software based on the morphology markers signal, and the ROIs were divided into 4 segments: DESMIN^+^ (DESMIN^+^MARCO^–^IBA1^–^), MARCO^+^ (DESMIN^–^MARCO^+^IBA1^+^), MARCO^–^ (DESMIN^–^MARCO^–^IBA1^+^), and OTHER (DESMIN^–^MARCO^–^IBA1^–^). RNA probes from each segment were subjected to library preparation and sequenced using Illumina NextSeq 2000. Two samples corresponded to CCl_4_-induced fibrosis plus adoptive transfer of MARCO^+^ BMDMs, and 2 were CCl_4_-induced fibrosis plus saline for 4 analyzed mice. Thirteen to 14 ROIs were selected on each liver sample; 7 of them were MARCO^hi^, and 6 or 7 were MARCO^lo^, with 216 segments (libraries). Raw data (BCL files) were converted to RNA-seq data (FASTQ files) with Illumina’s bcl2fastq v2.20; the FASTQs were processed with the GeoMx NGS Pipeline v3.1. After sequencing, reads were trimmed, merged, and aligned to a list of indexing oligos to identify the source probe. Each read’s Unique Molecular Identifier (UMI) region was used to remove PCR duplicates and duplicate reads, converting reads into digital counts. For DSP RNA expression evaluation, 203 segments were processed. Raw Illumina counts were normalized using the upper quartile (Q3), and standard QC threshold settings, as recommended by the manufacturer, were used to filter AOIs and probes. Low-quality samples were removed from analysis if the geometric mean of aligned reads from all probes was less than the geometric mean of the negative control probes; the Q3 of the counts in each segment was less than the geometric mean of the negative control probes in the data. Processing count data were done using GeoMxTools (v3.2) and NanoStringNCTools (v1.6).

Due to ambient hepatocyte gene contamination, additional steps were taken to refine the gene set and the ROIs. Genes primarily found in HSCs were identified via scRNA-Seq and compared against the filtered genes for matches, leaving 760 highly expressed HSC genes for analysis (Supplemental Figure 10B). The final number of segments for data processing was 159 after thresholding and QC filtering were performed.

Statistical analyses of the data generated here were performed using R (v4.1). Dimensionality reduction analysis was performed with principal components analysis to assess outliers and potential batch effects. Comparisons for individual gene expression were measured as a log_2_ fold-change (FC). A linear mixed model was used to fit the normalized RNA count data to identify differentially expressed (DE) genes. The adjustment method used to control the false discovery rate (FDR) for multiple testing used in this study was the Benjamini-Hochberg method. The analysis was carried out using the lme4 package (v1.1.31) and the stats package (v4.1) with emmeans (v1.8.4). Plotting outputs were done in ggplot2 (v 3.4.4).

### Statistics

All reported data represent at least 3 biological replicates. All continuous variables are summarized as mean ± SEM. Data were analyzed with a 1-way ANOVA and Tukey post hoc tests or 2-tailed *t* tests. All statistical analyses were performed with Prism GraphPad software version 10.1.2 for Windows (GraphPad Software; www.graphpad.com) except for IPA analyses, statistical tests were performed by IPA software using a right-tailed Fisher’s exact test to calculate *P* value. The threshold for statistical significance was set as *P* < 0.05.

### Study approval

All mouse in vivo protocols were approved by the Mayo Clinic IACUC, under protocol no. A00006669-24.

Formalin fixed-paraffin embedded 5 μm tissue sections of human liver from donors and from cirrhosis patients were obtained through the Mayo Center for Cell Signaling in Gastroenterology from samples collected at Mayo Clinic with institutional approval (IRB no. 15-008251) after receiving informed consent from patients.

### Data availability

All relevant data supporting the findings of this study are reported in the article. Transcriptomic data sets from scRNA-seq and spatial transcriptomics are available on the GEO database under GSE291711 and GSE291710. Transcriptomic data sets from Nanostring nCounter are available as supplementary material.

Additional methodology is available in the Supplemental Material. Supporting numerical data for each figure is available in the Supporting Data Values file.

## Author contributions

SJ, SAC, and SC contributed to the conceptualization of the study, experimental design, and data analysis. SJ and UY completed animal experiments and in vitro cell experiments. SJ, AAA, MFK, BA, MW, LCD, MHT, RDB, and NJS performed immunostaining, imaging, and in vitro experiments. SAC and YL performed in silico analysis for scRNA-seq. WAS, SJ, and SAC performed in silico analysis for spatial transcriptomics. EK contributed spatial transcriptomics data. EK, NJS, and MBH contributed to the manuscript draft conceptualization and revisions. SJ, SAC, and SC wrote the manuscript. SC and VHS contributed to the study design, resources, funding support, revision of the manuscript, and overall study supervision. SJ and SAC contributed equally and are considered co–first authors of this work. Co–first authorship was decided according to the amount of work dedicated to the experiments and manuscript, with SJ leading the design and development of the in vivo and in vitro experiments and with SAC leading the design and development of the in silico analysis.

## Funding support

This work is the result of NIH funding, in whole or in part, and is subject to the NIH Public Access Policy. Through acceptance of this federal funding, the NIH has been given a right to make the work publicly available in PubMed Central.

- R37AA021171 and R01DK59615 from the NIH (VHS)
- R01 DK136511 (EK) from the NIH
- CTSA Grant Number KL2 TR002379 from the National Center for Advancing Translational Science (NJS)
- PSC Partners (NJS)
- Mayo Clinic K2R Program (NJS)
- AASLD Foundation (NJS)
- Gilead Scholar Award (EK)
- Mayo Clinic Center for Cell Signaling in Gastroenterology Pilot and Feasibility Award P30 DK084567 (NJS)
- T32 DK124190 (SAC)
- Mayo Clinic Graduate School of Biomedical Sciences (SAC)
- Genome Analysis Core and Director, Stephen Murphy, PhD, supported by the Mayo Clinic’s Office of Core Shared Services and the Mayo Clinic Comprehensive Cancer Center Grant, funded by National Cancer Institute (P30CA15083)
- Microscopy Core and Epigenomics & Spatial Biology Core of the Mayo Clinic Center for Cell Signaling in Gastroenterology (National Institute of Diabetes and Digestive and Kidney Diseases of the NIH under award no. P30DK084567).

## Supplementary Material

Supplemental data

Supplemental data set 1

Unedited blot and gel images

Supporting data values

## Figures and Tables

**Table 1:**
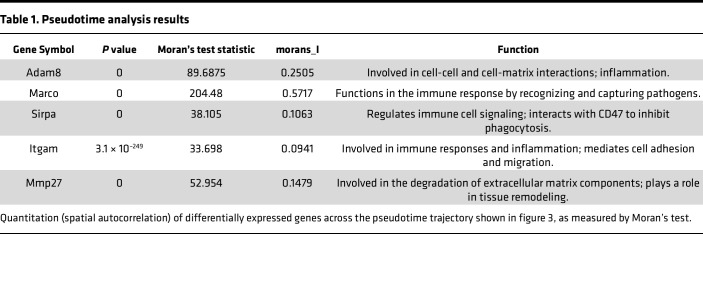
Pseudotime analysis results

## Version 1 03/23/2026

Electronic publication
