# Supplementary material for: Macrophages expressing macrophage receptor with collagen structure attenuate liver fibrosis through a tissue restoration phenotype
Source: JCI Insight. 2026 Mar 23;11(6):e193172. doi: 10.1172/jci.insight.193172 (PMC13043091; doi:10.1172/jci.insight.193172)
Supplement: Supplemental data [file jciinsight-11-193172-s330.pdf]

Macrophages expressing Macrophage Receptor with Collagen Structure Attenuate Liver Fibrosis Through a Tissue Restoration Phenotype

Supplemental Material

Methods

RAW 264.7 Macrophage Transfection and Migration Assay

The RAW 264.7 macrophage-like cell line (TIB-71, ATCC) was used for in vitro Marco expression experiments. RAW 264.7 cells were maintained at 37 °C and 5% CO2 in culture with Dulbecco modified eagle medium (11995-065; Gibco) supplemented with 10% FBS, 1% penicillin/streptomycin, and 10 mM HEPES (15630080, GIBCO). Cells were grown to a confluence of 60% to 70% and transfected with the pCMV3-C-GFPSpark Mouse Marco expression plasmid (MG50283-ACG; Sino Biological, Inc) or empty plasmid using FuGENE 4k (4k-1000; Fugene LLC) transfection reagent. After 24 hours, the transfected cells were seeded as a monolayer in a 35-mm tissue culture dish. A scratch was made in the cell monolayer by dragging a 10-μL pipette tip across the well bottom. Immediately after performing the scratch, the culture medium was replaced with fresh medium containing PBS as the vehicle or 10 ng/mL C-X-C motif chemokine ligand 9 (CXCL9) (578202, BioLegend) as a stimulant. Cells were imaged using Zeiss Axio-Observer 7 inverted microscope with live cell imaging over a 12-hour period using Zen software to track all the GFP+ cells and GFP- cells. The rate of cell migration was calculated by subtracting the scratch area after 12 hours from the scratch area at 0 hours and dividing the difference by the scratch area at 0 hours. The individual points in the graph represent individual cells. (27)

Reverse Transcription qPCR

We extracted mRNA from RAW 264.7 cells, bone marrow cells, and mouse liver with the RNeasy Mini Kit (74104; Qiagen). Total RNA (500 ng) was reverse transcribed with the SuperScript III First-Strand Synthesis System (18080-051; Invitrogen), and qPCR was performed with the iTaq Universal SYBR Green Supermix (1725120; Bio-Rad Laboratories, Inc) according to the manufacturer protocol. The primers used and sequences are listed in Supplemental Table 1.

Immunoblot Analysis

Liver tissues and BMDMs were lysed in RIPA lysis buffer (89900; Thermo Fisher Scientific) supplemented with protease inhibitors (11836170001; Roche). Protein concentrations were quantified with the Dc protein assay (5000116; Bio-Rad Laboratories, Inc). For each sample, 30 μg of total protein was loaded onto a polyacrylamide gel, subjected to electrophoresis with sodium dodecyl sulfate, and transferred to a nitrocellulose membrane. Membranes were blocked in 5% milk and incubated overnight with primary antibodies at 4 °C. Primary antibodies and dilutions are listed in Supplemental Table 2. Enhanced chemiluminescence (WBLUR0100; Millipore or sc-2048; Santa Cruz Biotechnology) was used for protein detection and immunoblots were visualized with a biomolecular imager (Azure Biosystems). Proteins were quantified with ImageJ software (26).

In vitro Phagocytosis Assay

Sorted MARCO-positive or MARCO-negative BMDMs were plated in 96-well plates at a density of 5,000 cells/well in 150 mL of RPMI supplemented with 10% FBS and incubated overnight at 37 °C in 5% CO2. The next day, the culture medium was removed, and the cells were incubated

in fresh RPMI with 1:200 fluorescein isothiocyanate–labeled rabbit immunoglobulin G–coated latex beads (500290; Cayman Chemical) for 2 hours at 37 °C. The cells were then washed, and fluorescence was measured with a GloMax microplate reader (Promega Corporation).

Immunostaining

Formalin fix-paraffin embedded 5-µm tissue sections of human liver from donors and from cirrhosis patients were obtained through the Mayo Center for Cell Signaling in Gastroenterology from samples collected at Mayo Clinic. Mouse livers from either the olive oil group or CCl_4 group mice were fixed in 10% formalin, embedded in paraffin, and sectioned into 5-µm sections.

Sections were deparaffinized, and heat-mediated antigen retrieval was performed in Tris-EDTA buffer (10mM Tris-base, 1mM EDTA, and 0.05% Tween 20; pH, 9.0). Sections were blocked in 10% FBS and 1% BSA for 1 hour at room temperature, followed by overnight incubation with primary antibodies. Primary antibodies and dilutions are listed in Supplemental Table 2.

For immunohistochemical analysis, slides were incubated with an appropriate biotinylated secondary antibody (Vector Laboratories Inc) for 1 hour at room temperature. The slides were then incubated with horseradish peroxidase–conjugated avidin reagent (PK-7100; Vector Laboratories Inc) for 30 minutes at room temperature. The slides were developed with 3,3′-diaminobenzidin substrate (SK-4100; Vector Laboratories Inc) for 3 minutes. Before mounting, the stained slides were counterstained with hematoxylin and dehydrated in ethanol and xylene.

For immunofluorescence staining, the slides were incubated with fluorophore-conjugated secondary antibodies for 1 hour and counterstained with DAPI for 3 minutes before mounting with antifade media. Images were acquired with a light microscope (ZEISS Microscopy),Evos Cell

Imaging System (Thermo Fisher Scientific), or LSM 780 laser scanning confocal microscope (ZEISS Microscopy).

Fluorescence-Activated Cell Sorting (FACS)

Cells were resuspended in FACS buffer (1% FBS, 0.1% BSA, and 2 mM EDTA in 1X PBS) and incubated with TruStain FcX PLUS (156604; BioLegend) to decrease nonspecific staining followed with conjugated primary antibodies (Supplemental Table 2). Sorting and data acquisition were performed with a FACSAria II SORP instrument and FACSDiva software, v8.0.1 (BD Biosciences). Sorting was based on manual gating. The results were analyzed with FlowJo software, v10.10.0 (FlowJo, LLC).

Mass cytometry by Time-of-flight (CyTOF)

Intrahepatic leukocytes were isolated as described in the single cell RNA seq methodology section. 3 million cells were fixed and stained with a macrophage focused antibody cocktail (Supplementary Table 3) conjugated to lanthanide-based metal isotopes according to manufacturer guidelines (Fluidigm, San Francisco, CA). Mass cytometry was performed at the Mayo Clinic Immune Monitoring Core (Mayo Clinic, Rochester, MN) on a Helios mass cytometry system (Fluidigm, San Francisco, CA). Signal normalization was achieved by spiking EQ™ four element calibration beads (Cat. No. 201078, Fluidigm, San Francisco, CA) into each sample. Data normalization was performed using CyTOF software version 6.7.1014 (Fluidigm). FlowJo Software (version 10.8.1; Becton, Dickinson & Company) was used to identify cell singlets ($^{191}Ir^{+193}Ir^{+}$) and viable events (^{195} Pt^{-}) before performing high dimensional data analysis. All live

singlet events were exported to new ".fcs" (flow cytometry standard) files prior to analysis. t-SNE clustering analysis was performed on 10,000 equivalent events from each sample with selection of all parameters. Cellular phenotypes were assigned to the t-SNE plot based on distribution and expression characteristics of all markers after clustering. Phenographs were generated in the R language-based Cytofkit software[1].

Hydroxyproline Content Measurement

Hydroxyproline content was quantified using a colorimetric assay test kit (#MAK569, Millipore-Sigma) following manufacturer instructions. 10 mg of liver tissue were homogenized in 100 µL of ultrapure water. 100 µL of pure Hydrochloric Acid were added to the homogenized tissue and were incubated for hydrolyzation at 120 degrees Celsius for 3 hours. Later, 50 µL of hydrolyzed material were dried at 60 degrees Celsius overnight. The dry material was resuspended in 100 µL of Chloramine T/Oxidation Buffer followed by addition of 100 µL of DMAB Reagent in perchloric acid. After 90 minutes incubation at 60°C, the absorbance was measured at 560 nm. Hydroxyproline concentration was quantified with a standard curve using a hydroxyproline standard provided by in the kit.

Supplemental Tables

Supplemental Table 1. Antibodies used in the study

Target	Species reactivity	Conjugation	Dilution	Technique	Catalog #	Clone	Company
CD68	Human	None	1:100	IF, IHC	Ab213363	EPR20545	Abcam
CD68	Human	None	1:100	IF	MAB20401	298807	Bio-Techne
CD68	Mouse	None	1:200, 1:1000	IF, IHC, WB	Ab125212	Polyclonal	Abcam
CD68	Mouse	None	1:100	IF	Ab201340	C68/684	Abcam
CD11b	Mouse	VioGreen	1:200	FC	130-113-811	M1/70.15.11.5	Miltenyi
CD45	Mouse	VioBlue	1:200	FC	130-110-802	30F11	Miltenyi
CLEC4f	Mouse	None	1:100	IF, IHC	AF2784	Polyclonal	Bio-Techne
Collagen I	Human, Mouse	None	1:200	IF, WB	1310-01	Polyclonal	Southern Biotechnologies
F4/80	Mouse	APC	1:100	FC	123116	BM8	BioLegend
F4/80	Mouse	PerCP-Vio700	1:200	FC	130-118-466	BM8	Miltenyi
F4/80	Mouse	None	1:200	IF	70076s	D2S9R	Cell Signaling Technologies
GFP	N/A	None	1:100	IF	55494S	5G4	Cell Signalling Technologies
MARCO	Human	None	1:100	IF, IHC	PA5-64134	Polyclonal	Invitrogen
MARCO	Mouse	PE	2 ug/mL	FC	FAB29561P	2359A	Novus Biologicals
MARCO	Mouse	None	1:200	IF	AF2956	Polyclonal	Bio-Techne
MARCO	Mouse	None	1:100 1:1000	IF, IHC, WB	ab239369	EPR22944-64	Abcam
IBA1	Mouse	Alexa Fluor 647	1:200	IF	78060	E4O4W	Cell Signaling Technologies
DESMIN	Mouse	None	1:50	IF	14-9747-82	DE-U-10	Invitrogen
GAPDH	Mouse	None	1:5000	WB	MA5-15738	GA1R	Invitrogen
IgG	Goat	Alexa fluor 594	1:300	IF	A11058	Polyclonal	Invitrogen

IgG	Goat	Alexa fluor 488	1:300	IF	A11055	Polyclonal	Invitrogen
IgG	Goat	Alexa fluor 568	1:300	IF	A11057	Polyclonal	Invitrogen
IgG	Goat	Alexa fluor 647	1:300	IF	A21447	Polyclonal	Invitrogen
IgG	Mouse	Alexa fluor 488	1:300	IF	A21202	Polyclonal	Invitrogen
IgG	Mouse	Alexa fluor 568	1:300	IF	A10037	Polyclonal	Invitrogen
IgG	Mouse	Alexa fluor 647	1:300	IF	A31571	Polyclonal	Invitrogen
IgG	Rabbit	Alexa fluor 488	1:300	IF	A21206	Polyclonal	Invitrogen
IgG	Rabbit	Alexa fluor 568	1:300	IF	A10040	Polyclonal	Invitrogen
IgG	Rabbit	Alexa fluor 647	1:300	IF	A31573	Polyclonal	Invitrogen
IgG	Rat	Alexa fluor 647	1:300	IF	A21208	Polyclonal	Invitrogen

Supplemental Table 2. Mouse qPCR primer sequences

Gene	Forward sequence	Reverse sequence
MMP9	CCGACTTTTGTGGTCTTCCCC	CTCTCCCATCATCTGGGCGG
MMP12	AGCTGCACTCTGCTGAAAGG	AGCCCCACAGGCAGATACC
CCL5	CCTGCTGCTTTGCCTACCTC	CACACACTTGGCGGTTCCTT
TNF	CCACGTCGTAGCAAACCAAA	GACAAGGTACAACCCATCGGC
ACTB	CCTCCCTGGAGAAGAGCTATG	TTACGGATGTCAACGTCACAC
MARCO	CTTCTGTCGCATGCTCGGTT	AGATGTTCCCAGAGCCACCT
IL10	GTGGAGCAGGTGAAGAGTGA	TCGGAGAGAGGTACAAACGAG
CXCR3	AGCCATGTACCTTGAGGTTAGTGA	CAGGCTGAAATCCTGTGGGC
MMP3	TATACGAGGGCACGAGGAGC	TCTTCACGGTTGCAGGGAGA
MMP2	GGACAAGTGGTCCGCGTAAA	CCGACCGTTGAACAGGAAGG

Supplemental Table 3. Mouse Macrophage Antibody Panel for CyTOF

Target	Label	Clone	Localization
CD45	089Y	30-F11	Surface
CD44	106Cd	IM7	Surface
CD38	110Cd	90	Surface
CD169/Siglec-1	112Cd	QA20A47	Surface
MARCO	114Cd	2359A	Surface
CD36	116Cd	HM36	Surface
Lgals3	141Pr	202213	Surface
CD204	143Nd	1F8C33	Surface
MHC Class I	144Nd	28-14-8	Surface
CD69	145Nd	H1.2F3	Surface
PD-L1	146Nd	10F.9G2	Surface
CD9	147Sm	MZ3	Surface
CD80	148Nd	16-10A1	Surface
Tim4	149Sm	370901	Surface
I-A/I-E	150Nd	M5/114.15.2	Surface
CD206 (MMR)	151Eu	C068C2	Surface
CLEC4F/CLECSF13	153Eu	poly Goat	Surface
CD16/CD32	154Sm	93	Surface
MERTK	155Gd	108928	Surface

CCR2	156Gd	475301	Surface
CD86	158Gd	GL1	Surface
F4/80	159Tb	BM8	Surface
CD64	160Gd	290322	Surface
Ly6G	161Dy	1A8	Surface
CCR5	163Dy	225307	Surface
CX3CR1	164Dy	SA011F11	Surface
CD14	165Ho	Sa14-2	Surface
CD19	166Er	6D5	Surface
TREM2	167Er	237920	Surface
CD8a	168Er	53-6.7	Surface
TRAIL	169Tm	N2B2	Surface
CD11b (Mac-1)	172Yb	M1/70	Surface
VSIG4	173Yb	poly Goat IgG	Surface
CD115/CSF1R	174Yb	AFS98	Surface
Ly6C	175Lu	HK1.4	Surface
CD45R (B220)	176Yb	RA3-6B2	Surface
CD3e	196Pt	145-2C11	Surface
CD11c	209Bi	N/A	Surface

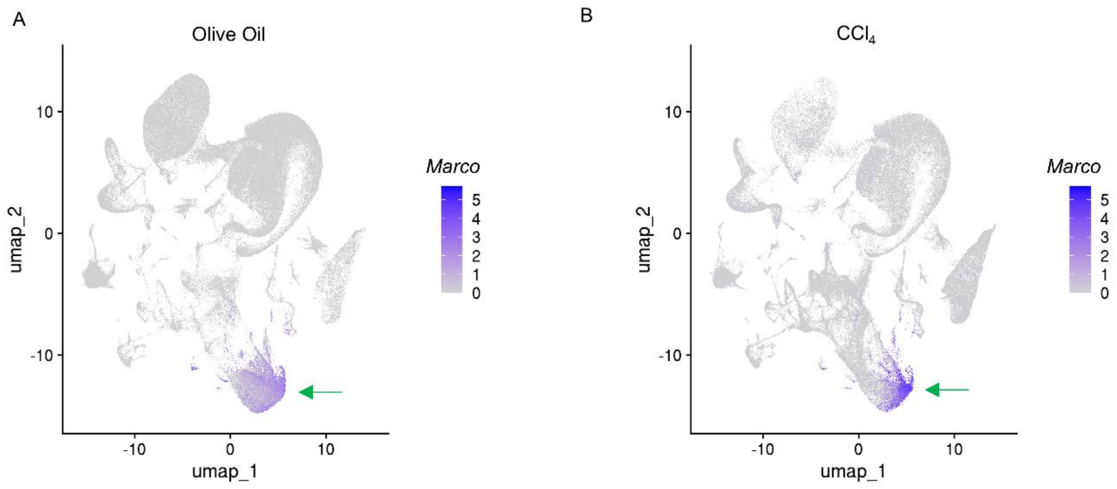

Supplemental Figure 1. Feature Plots of *Marco* Expression between Macrophages derived from CCl_4-induced chronic fibrosis and Olive Oil Controls. Feature plots split by group to highlight that while endogenous expression of *Marco* is low in the healthy olive oil administered liver **(A)**, it is detectable by scRNAseq and shown to clearly increase with CCl_4 chronic fibrotic injury **(B)**.

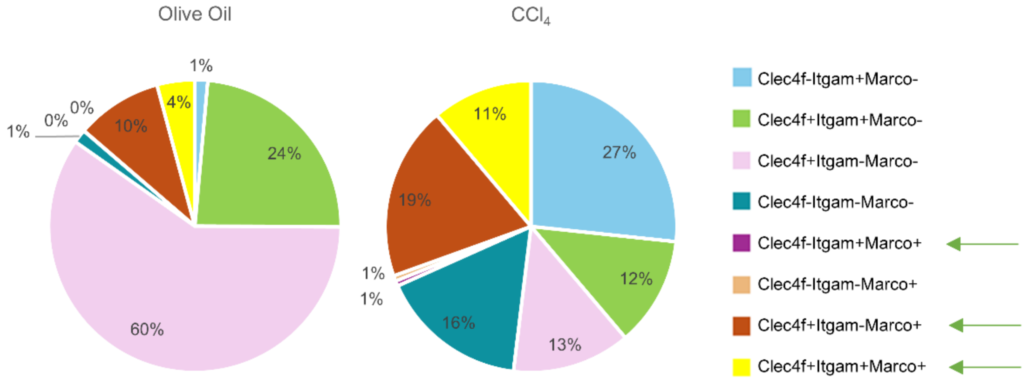

Supplemental Figure 2. Bioinformatic Sorting Demonstrates Cell Subset Changes in chronic CCl_4 -induced fibrosis and populations of *Marco*-positive, *Clec4f*-negative cells. Proportions of total macrophages positive for transcripts of cell markers *Clec4f* (Kupffer cells), *Itgam* (infiltrating macrophages), and *Marco* are shown with green arrows. The compartment of cells positive for *Marco* expands substantially with CCl_4-induced fibrosis.

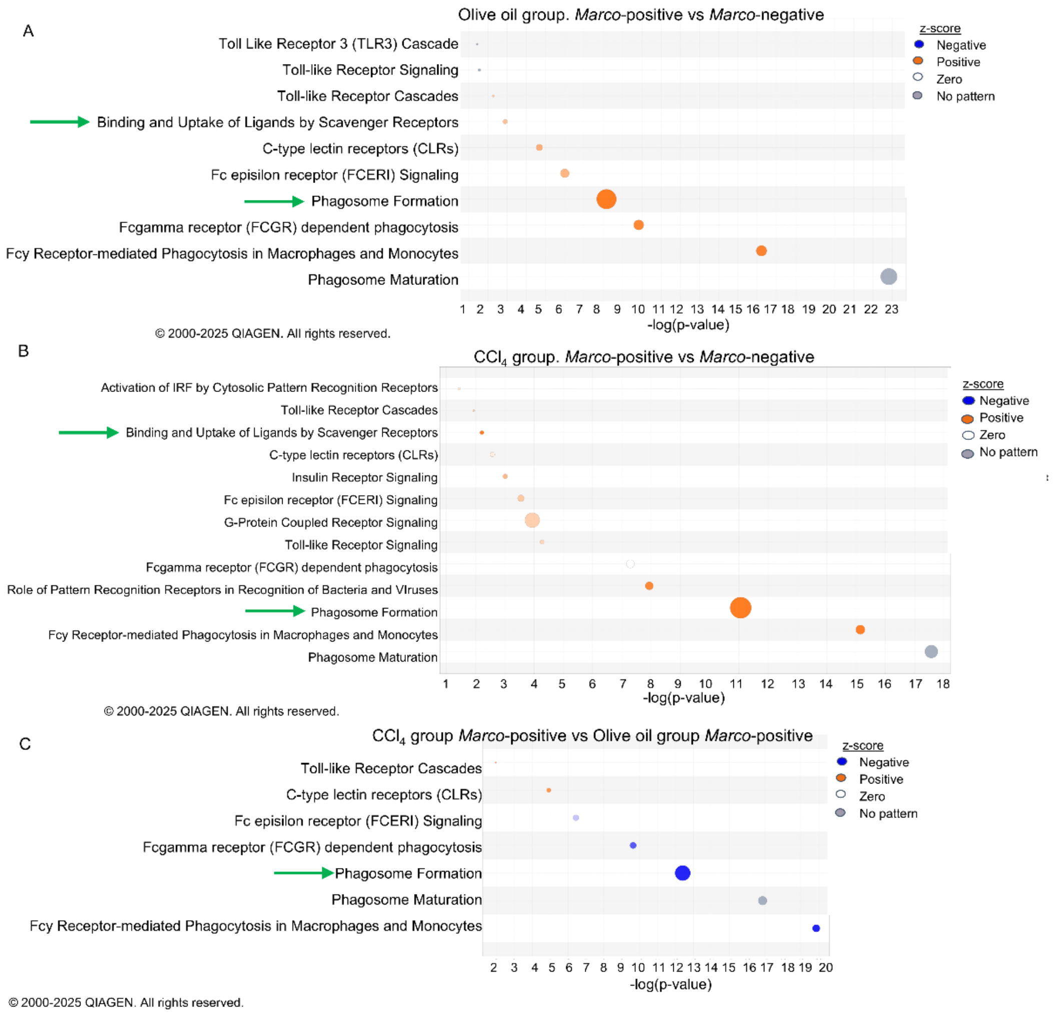

Supplemental Figure 3. Potential Phagocytic Differences between *Marco*-positive cells derived from Healthy Olive Oil vs CCl_4 injured murine livers by IPA of scRNAseq. (A) IPA of *Marco*-positive versus *Marco*-negative macrophages from the healthy olive oil control murine liver highlights upregulation of phagosome formation pathways (green arrow) indicating potential increases in phagocytosis. **(B)** IPA of *Marco*-positive versus *Marco*-negative macrophages from CCl_4 injured murine liver. **(C)** IPA of *Marco*-positive macrophages from CCl_4 injured murine liver versus healthy liver. Notably, downregulated pathways show features impaired in *Marco*-positive macrophages induced in an injury microenvironment versus endogenous *Marco*-positive macrophages in healthy liver. Results are filtered to phagocytosis relevant pathways.

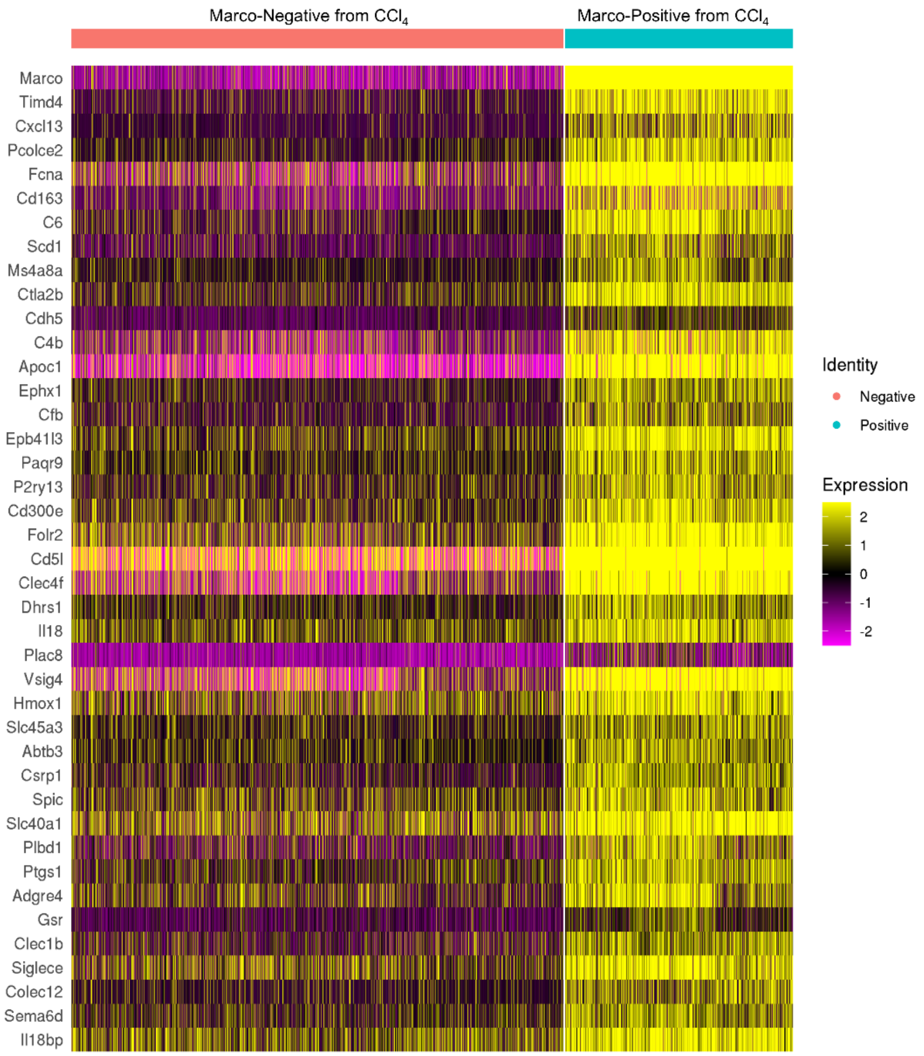

Supplemental Figure 4. Heatmap of top differentially expressed genes between *Marco*-positive vs *Marco*-negative macrophages within CCl_4-injured livers. Macrophages were bioinformatically sorted and differential gene expression run for *Marco*-positive versus *Marco*-negative macrophages solely within the CCl_4 injured liver sample to isolate specific changes within *Marco*-positive cells from the fibrotic microenvironment.

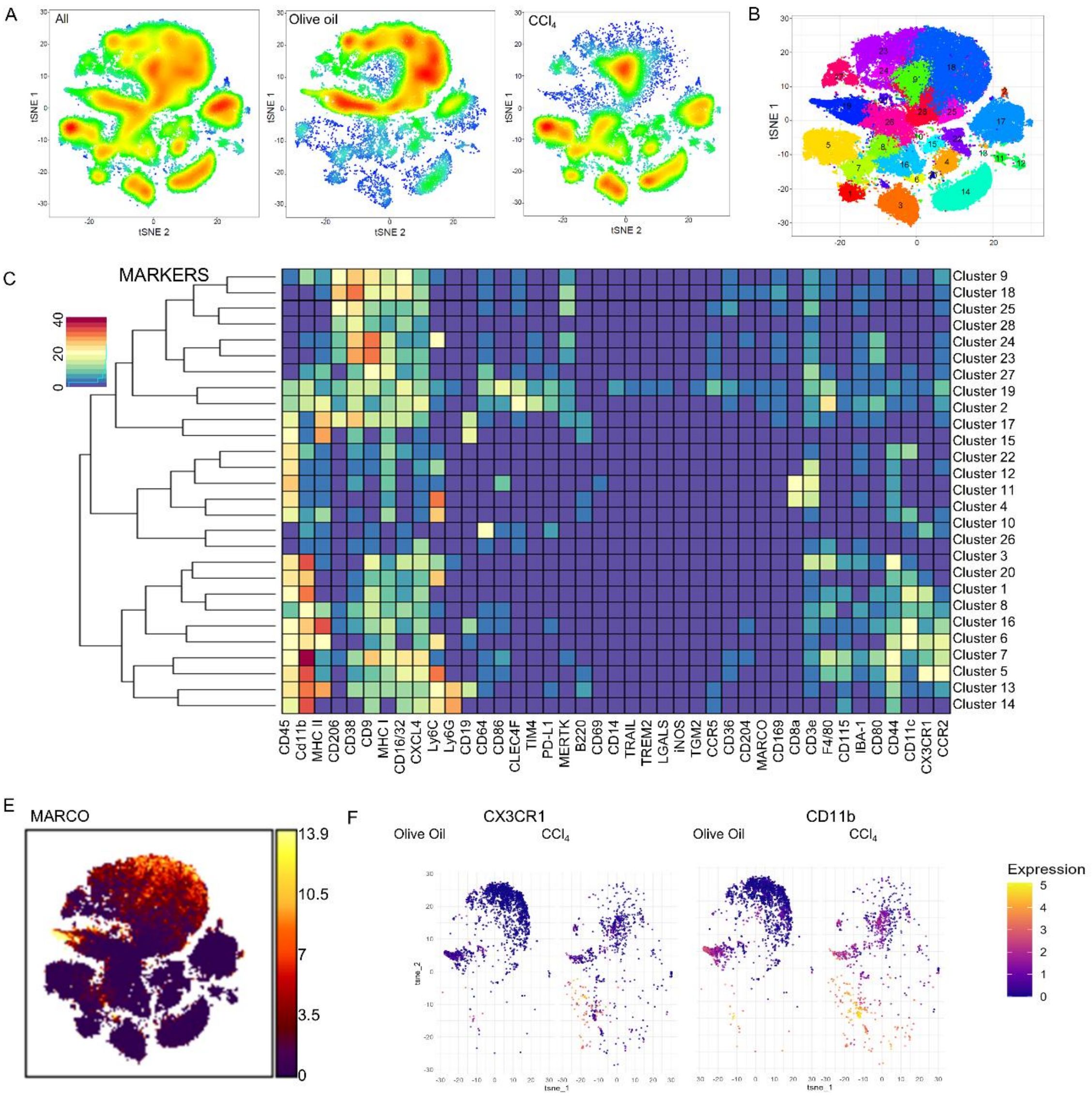

Supplemental Figure 5. Mass cytometry analysis of intrahepatic leukocytes isolated from control (Olive oil) or fibrotic (CCl_4) mouse livers. Cluster heterogeneity changes can be observed in control and fibrotic intrahepatic leukocytes **(A).** A total of 28 individual clusters were identified across all of the samples analyzed **(B).** The marker median distributions across clusters are shown in panel **(C).** MARCO-positive cells are distributed across several main clusters **(D).** Cells were sorted based on MARCO expression, and the top 10% were subset to analyze the expression of infiltrating macrophage markers CX3CR1 **(E)** and CD11b **(F).**

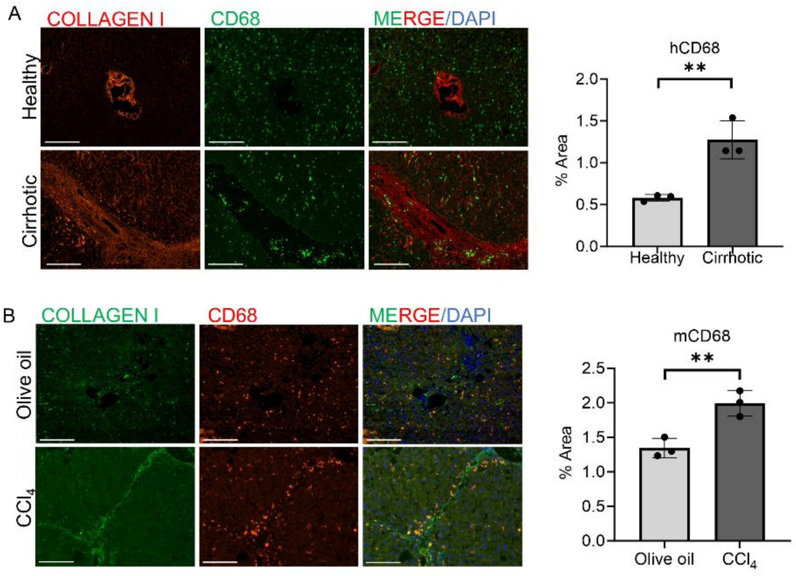

Supplemental Figure 6. **The Macrophage Population Is Increased in the Chronic Fibrotic Liver. (A)** Representative immunofluorescence images of CD68 (macrosialin) staining for macrophages and collagen type I in human liver tissue sections from patients with and without cirrhosis and quantification of CD68 as % of stained area. **(B)** Representative immunofluorescence images of CD68 and collagen type I in liver tissue sections from mice after olive oil or CCl_4-administration, quantification of CD68 percentage of stained area. ***P*<0.01. Scale bar: 275µm. CCl_4 indicates carbon tetrachloride; CD68, macrosialin; DAPI, 4′6-diamidino-2-phenylindole.

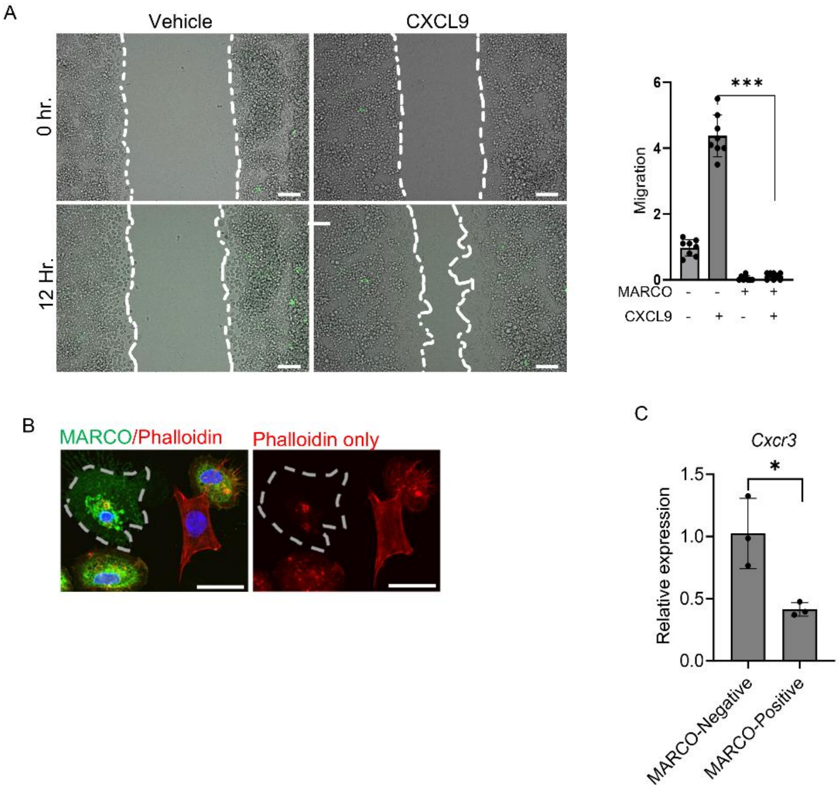

Supplemental Figure 7. LSEC-Derived CXCL9 Promotes MARCO-Negative Macrophage Migration in vitro through CXCR3. (A) Representative still images from real-time video microscopy analysis of migrating RAW 264.7 cells overexpressing MARCO (green) versus basal MARCO-negative (clear) after CXCL9-induced chemotaxis for 12 hours. Scale bar = 100 μm **(B)** Representative fluorescence images of MARCO and phalloidin co-staining in RAW 264.7 cells. Scale bar = 10 μm. **(C)** Expression of C-X-C chemokine receptor type 3 (*Cxcr3*) mRNA in MARCO-positive and MARCO-negative macrophages by qPCR. ***P<0.001, *P<0.05. CCl_4 indicates carbon tetrachloride; CXCL9, C-X-C motif chemokine 9; DAPI, 4′6-diamidino-2-phenylindole; LSEC, liver sinusoidal endothelial cell; MARCO, macrophage receptor with collagenous structure.

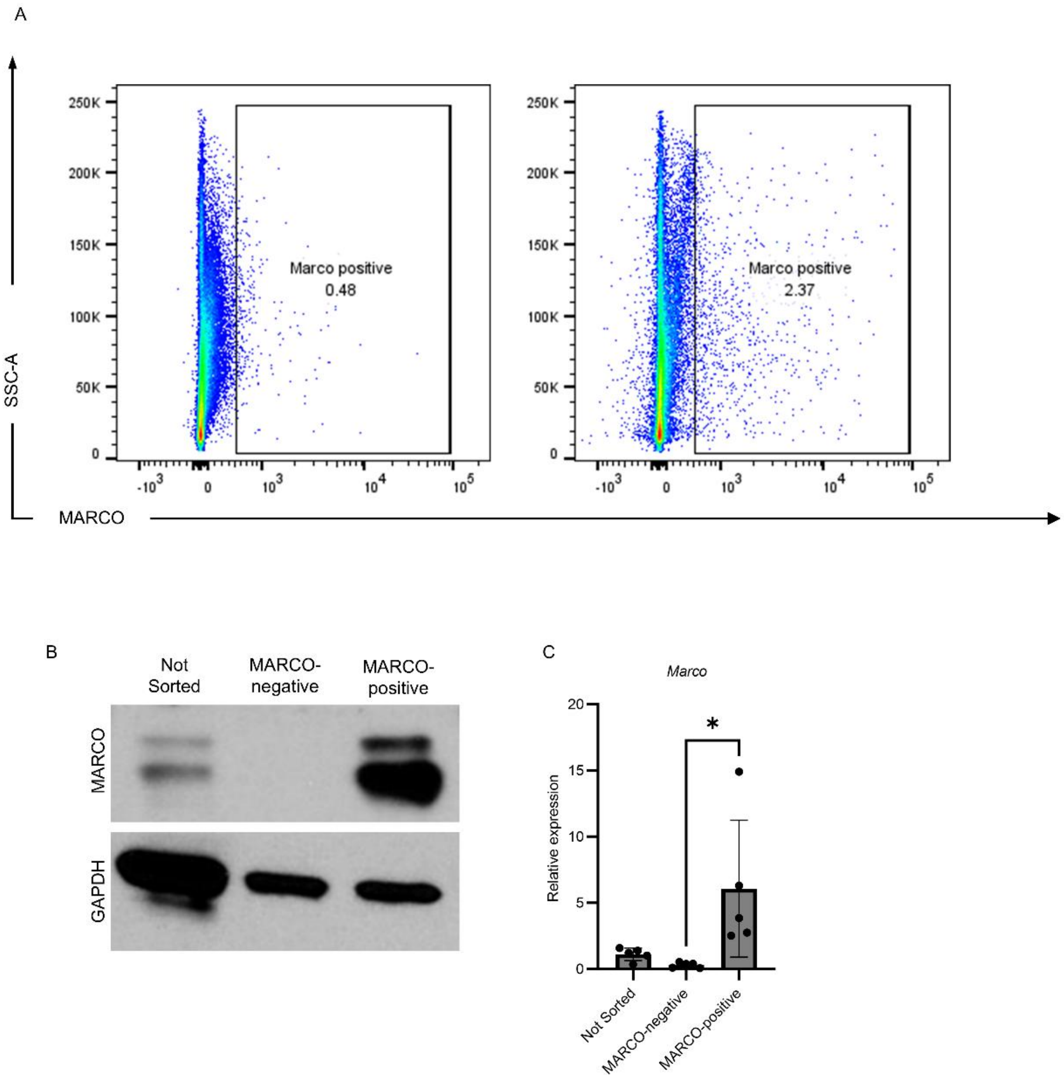

Supplemental Figure 8. ***Marco*** **expression in intrahepatic macrophages versus induction in BMDM and detection after sorting. (A)** Intrahepatic macrophage isolation from olive oil (left) and CCl_4 (right), followed by flow cytometry sorting to obtain *Marco*-positive cells, yields far too few cells to be useful for translational applications or for in vitro study. To work around this issue, LPS stimulated BMDMs expressing MARCO protein **(B)** and mRNA **(C)** were used for Figure 5 in vitro and Figures 6 and 7 in vivo studies.

A

Top Upstream Regulators

Upstream Regulators

Name	p-value	Predicted Activation
lipopolysaccharide	9.29E-97	Activated
Immunoglobulin	5.97E-72	
IFNG	5.50E-60	
NFE2L2	3.98E-50	Activated
CSF1	3.64E-47	

1 2 3 4 5 6 7 8 9 >

B

Top findings from Ingenuity Knowledge Base (show all 51279 categorized literature findings

Regulates:	TNF, IL6, IL1B, NOS2, PTGS2, IL10, CCL2, IL12B, CXCL8, NFkB (complex), TLR4, CCL5, nitric oxide, IFNB1, IFNG
Regulated by:	LBP, TLR4, CD14, lipopolysaccharide, LY96, CAMP, ethanol, Casp4, AOAH, BPI, Immunoglobulin, TLR, HMGB1, SCARB1, APOA1
Binds:	TLR4, CD14, LBP, LY96, TLR2, HMGB1, TLR, HDL, APCS, BPI, CAMP, LTF, hemoglobin, IgG, ITGAM
Role in cell:	expression in, production in, phosphorylation in, activation in, activation, response, binding in, apoptosis, degradation in, proliferation

C

lipopolysaccharide 1

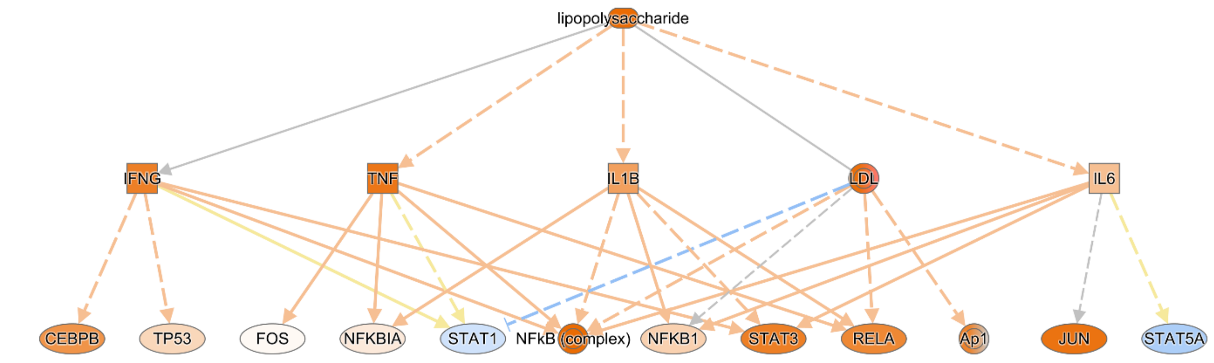

© 2000-2025 QIAGEN. All rights reserved.

Supplemental Figure 9. IPA Upstream regulator analysis of *Marco*. (A) *Marco*-positive macrophages from CCl_4-induced fibrotic livers reveals LPS as an upstream regulator of *Marco*. This agrees with the literature and practice of using LPS to generate *Marco*-positive BMDM **(B)** Molecular targets of LPS and LPS signaling per the IPA database. **(C)** IPA mechanistic network for LPS showing potential intermediate signaling partners responsible for *Marco* induction.

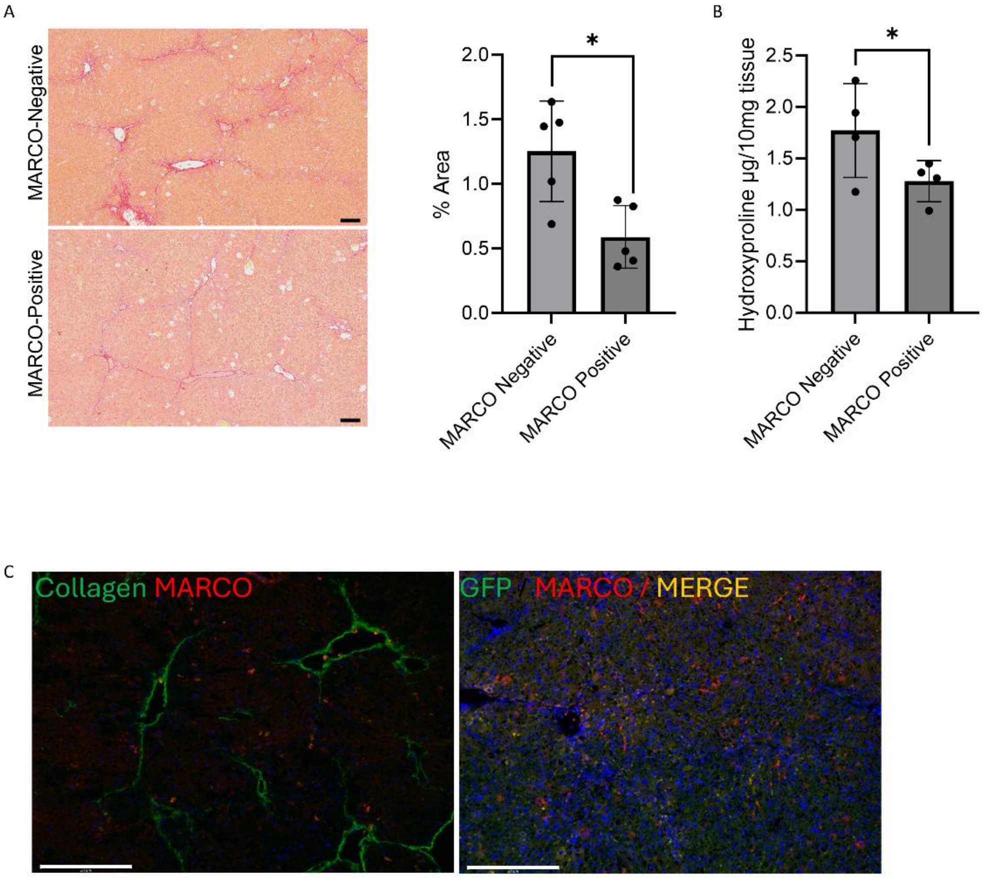

Supplemental Figure 10. **Macrophage Depletion with Clodronate followed by Adoptive Transfer of MARCO-positive macrophages decreases CCl_4 induced fibrosis. (A)** Clodronate containing liposomes were used to deplete endogenous macrophages 48 hours prior to injection with either MARCO-positive or MARCO-negative BMDMs and demonstrate the specific contribution of the transferred BMDMs in the reduction in fibrosis by Picrosirius Red staining. Scale bar = 100 µm **(B)** Quantification of Picrosirius Red Staining shows a significant decrease in fibrosis with transfer of MARCO-positive macrophages into a clodronate-induced macrophage-depleted background with CCl_4 injury. *($P < 0.05$). **(C)** Immunofluorescence staining showing collagen I accumulation in a sample of live cells from a mouse with CCl4-induced fibrosis treated with MARCO-positive/GFP-positive BMDM. Scale bar = 275 µm.

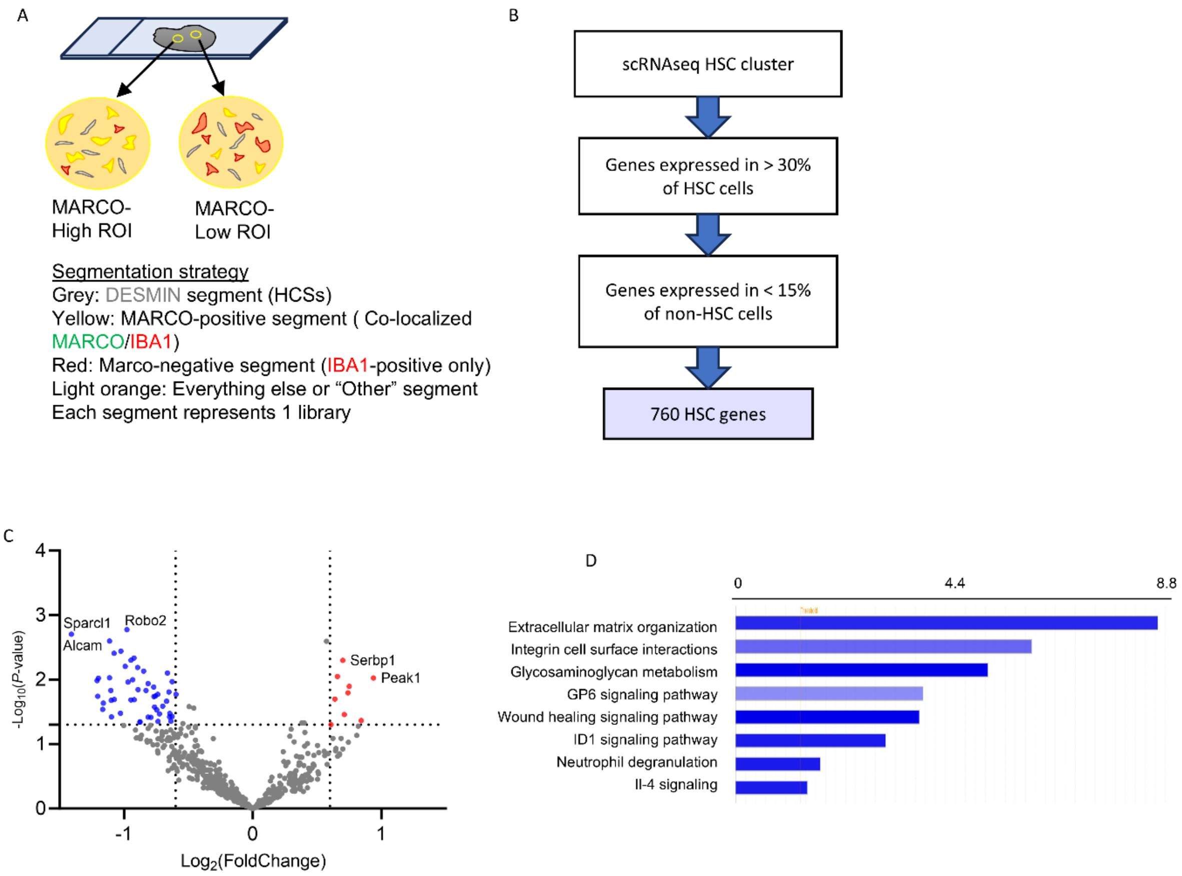

Supplemental Figure 11. Spatial transcriptomic analysis. (A) Segmentation strategy. **(B)** Strategy to subset the dataset to focus the analysis on HSC-expressed genes using our scRNAseq data set as reference. **(C)** Volcano plot of HSC differential gene expression in DESMIN-positive MARCO-High versus MARCO-Low areas with CCl_4-induced fibrosis and no exogenous MARCO-positive BMDM transfer. **(D)** Ingenuity pathway analysis results show dysregulated pathways in HSC from MARCO-High ROIs compared to MARCO-Low ROIs.

Reference

1. Chen H, Lau MC, Wong MT, Newell EW, Poidinger M, Chen J. Cytofkit: A Bioconductor Package for an Integrated Mass Cytometry Data Analysis Pipeline. *PLoS Comput Biol*. Sep 2016;12(9):e1005112. doi:10.1371/journal.pcbi.1005112
