# Supplementary figures and images for: Macrophages expressing macrophage receptor with collagen structure attenuate liver fibrosis through a tissue restoration phenotype
Source: JCI Insight. 2026 Mar 23;11(6):e193172. doi: 10.1172/jci.insight.193172 (PMC13043091; doi:10.1172/jci.insight.193172)

Full unedited gel Figure 6C

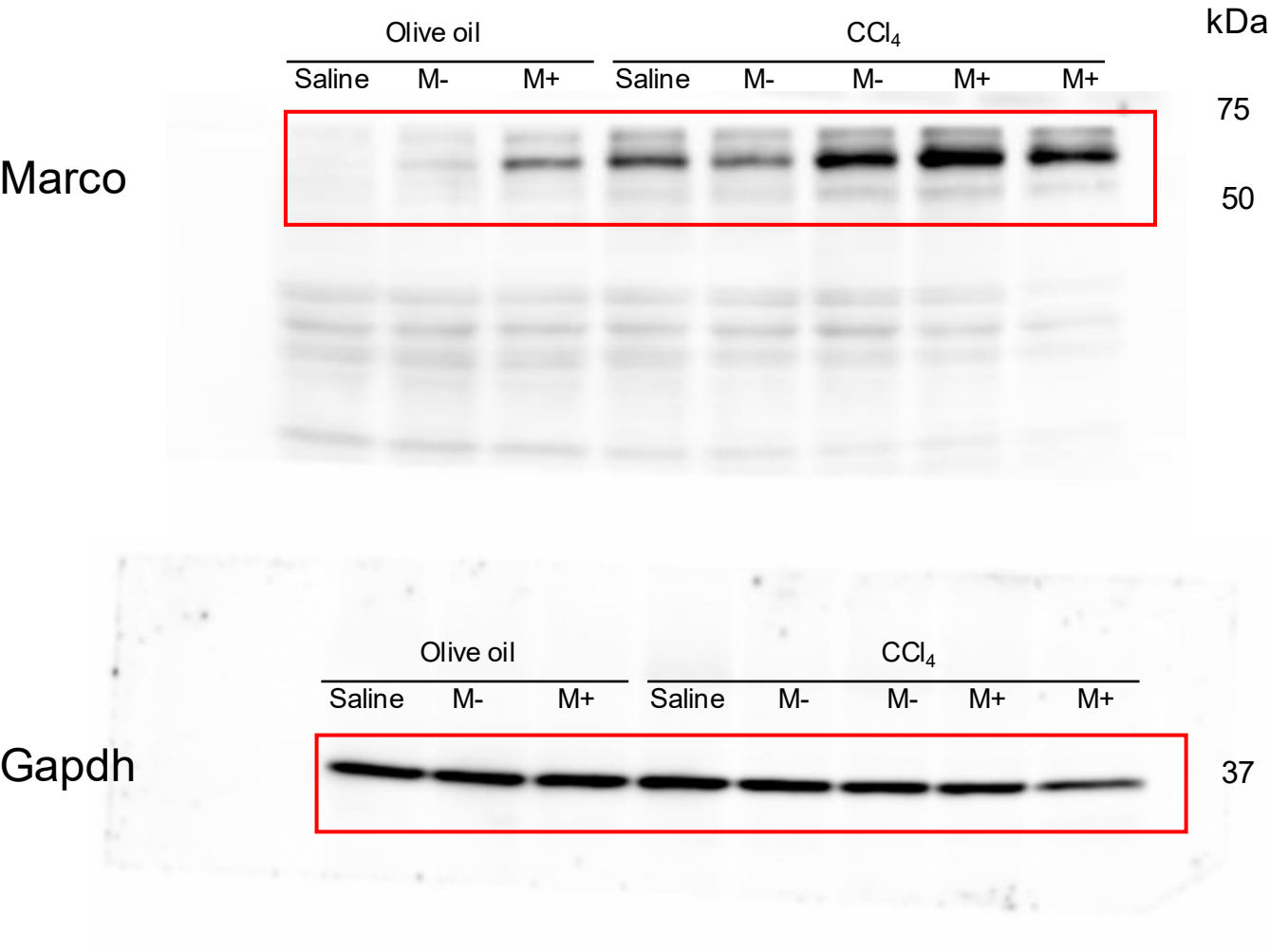

Full unedited gel Supplemental Figure 8B

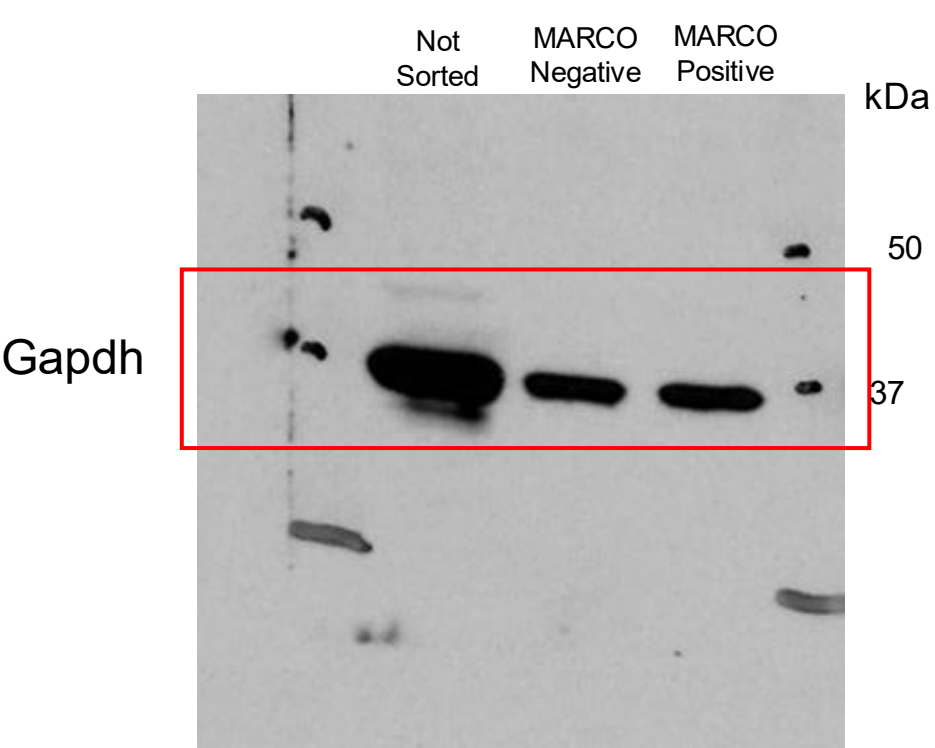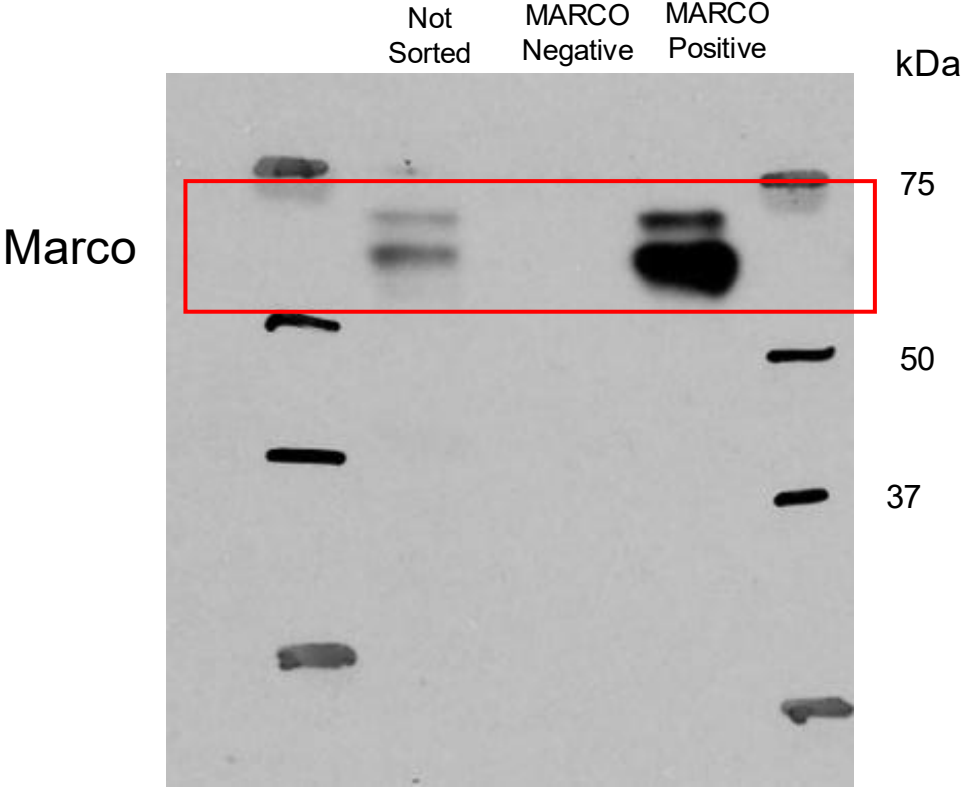

Supplement: Unedited blot and gel images [file jciinsight-11-193172-s332.pdf]
